# POPDC1 scaffolds a complex of adenylyl cyclase 9 and the potassium channel TREK‐1 in heart

**DOI:** 10.15252/embr.202255208

**Published:** 2022-10-18

**Authors:** Tanya A Baldwin, Yong Li, Autumn N Marsden, Susanne Rinné, Anibal Garza‐Carbajal, Roland F R Schindler, Musi Zhang, Mia A Garcia, Venugopal Reddy Venna, Niels Decher, Thomas Brand, Carmen W Dessauer

**Affiliations:** ^1^ Department Integrative Biology and Pharmacology McGovern Medical School, University of Texas Health Science Center Houston TX USA; ^2^ Institute for Physiology and Pathophysiology, Vegetative Physiology and Marburg Center for Mind, Brain and Behavior MCMBB Philipps‐University of Marburg Marburg Germany; ^3^ National Heart and Lung Institute, Imperial College London London UK; ^4^ Department Neurology McGovern Medical School, University of Texas Health Science Center Houston TX USA

**Keywords:** ADCY9, adenylyl cyclase, BVES, heart rate variability, TREK‐1, Cardiovascular System, Membranes & Trafficking, Signal Transduction

## Abstract

The establishment of macromolecular complexes by scaffolding proteins is key to the local production of cAMP by anchored adenylyl cyclase (AC) and the subsequent cAMP signaling necessary for cardiac functions. We identify a novel AC scaffold, the Popeye domain‐containing (POPDC) protein. The POPDC family of proteins is important for cardiac pacemaking and conduction, due in part to their cAMP‐dependent binding and regulation of TREK‐1 potassium channels. We show that TREK‐1 binds the AC9:POPDC1 complex and copurifies in a POPDC1‐dependent manner with AC9 activity in heart. Although the AC9:POPDC1 interaction is cAMP‐independent, TREK‐1 association with AC9 and POPDC1 is reduced upon stimulation of the β‐adrenergic receptor (βAR). AC9 activity is required for βAR reduction of TREK‐1 complex formation with AC9:POPDC1 and in reversing POPDC1 enhancement of TREK‐1 currents. Finally, deletion of the gene‐encoding AC9 (*Adcy9)* gives rise to bradycardia at rest and stress‐induced heart rate variability, a milder phenotype than the loss of *Popdc1* but similar to the loss of *Kcnk2* (TREK‐1). Thus, POPDC1 represents a novel adaptor for AC9 interactions with TREK‐1 to regulate heart rate control.

## Graphical abstract

Adenylyl cyclase type 9 (AC9) regulates resting heart rate and binds the novel scaffolding protein POPDC1. The two‐pore potassium channel TREK‐1 is released in a cAMP‐dependent manner from a POPDC1:AC9 complex to regulate TREK‐1 potassium currents.

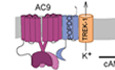

## Introduction

Cyclic AMP mediates the sympathetic regulation of many physiological functions in the heart, including contractility, relaxation, heart rate (HR), conduction velocity, and stress responses (Baldwin & Dessauer, 2018). The robust and specific control of numerous activities by cAMP within a single chamber and/or nodal cardiomyocyte is due not only to the diversity of adenylyl cyclase (AC) isoforms and cAMP effector molecules but also to the spatial control of cAMP signaling by the generation of macromolecular complexes (Baldwin & Dessauer, 2018; Musheshe *et al*, 2018; Marsden & Dessauer, 2019). These complexes are organized by a family of A‐kinase anchoring proteins (AKAPs) that place protein kinase A (PKA) in close proximity to its targets for regulation by phosphorylation (Omar & Scott, 2020). Many of these complexes also contain AC and phosphodiesterase, providing a framework for integration and modulation of local cAMP signaling within distinct AC‐PKA protein complexes or nanodomains (Scott *et al*, 2013; Ahmad *et al*, 2015). There are 9 transmembrane AC isoforms that display diverse patterns of regulation, yet only a subset of AC isoforms binds to an individual AKAP (Dessauer, 2009; Johnstone *et al*, 2018). For example, coupling AC5 with a downstream effector of PKA (i.e., TRPV1) on AKAP79 can sensitize this anchored effector to local cAMP production, reducing the IC_50_ for agonists by 100‐fold (Li *et al*, 2012, 2019; Efendiev *et al*, 2013). Scaffolding of AC9 to AKAP9 (a.k.a. Yotiao) promotes the PKA‐dependent phosphorylation of KCNQ1 and is required for regulation of the I_Ks_ current in cardiomyocytes (Li *et al*, 2012, 2019).

We show herein that the Popeye domain‐containing (POPDC) protein represents a novel scaffolding protein for AC isoforms. POPDC isoforms 1–3 were named for their conserved and abundant expression in skeletal and cardiac muscle and are important for cardiac pacemaking and conduction (Schindler *et al*, 2016a). POPDC proteins are heavily glycosylated, containing a short amino‐terminal extracellular domain, three transmembrane domains, and a cytosolic Popeye domain that displays structural similarity to the regulatory subunit of PKA and binds cAMP with high (~ 120 nM) affinity (Froese *et al*, 2012). Loss of either *Popdc1* (a.k.a. *Bves*) or *Popdc2* in mice or zebrafish results in arrhythmic and bradycardic phenotypes with high HR variability (Froese *et al*, 2012; Kirchmaier *et al*, 2012; Schindler *et al*, 2016b), while human mutations of POPDC family members cause limb‐girdle muscular dystrophy (LGMD), cardiac arrhythmia, familial atrioventricular (AV) block, and are implicated in long‐QT syndrome and heart failure (Gingold‐Belfer *et al*, 2011; Tan *et al*, 2013; Wang *et al*, 2016; Schindler *et al*, 2016b; Brand, 2019; Han *et al*, 2019; Vissing *et al*, 2019; Indrawati *et al*, 2020; Rinné *et al*, 2020). POPDC1 and POPDC2 bind the two‐pore‐domain potassium channel TREK‐1 (KCNK2 or K2P2.1) to enhance channel density at the plasma membrane and increase K^+^ currents (Froese *et al*, 2012; Schindler *et al*, 2016b; Rinné *et al*, 2020). Upon cAMP binding to POPDC proteins, the POPDC:TREK‐1 complex is dissociated (Froese *et al*, 2012; Rinné *et al*, 2020); loss of this regulation is proposed in part to give rise to HR variability upon deletion of *Popdc1* or *‐2*.

AC9 regulates HR as well (Li *et al*, 2017). We show herein that deletion of *Adcy9* gives rise to a bradycardia at rest and HR variability during recovery from stress, albeit with a milder phenotype than the loss of *Popdc1* or *Popdc2* (Froese *et al*, 2012). AC9 binds all three POPDC isoforms, interacting with both the transmembrane regions and the cytosolic Popeye domain of POPDC1. TREK‐1 co‐localizes and associates with the AC9:POPDC complex, while the deletion of *Adcy9* or *Popdc1* reduces TREK‐1‐associated Gαs‐stimulated AC activity in heart. TREK‐1 also interacts with the calcium calmodulin‐stimulated AC isoforms (AC1 and AC8); however, this interaction does not require POPDC1. Binding of AC9 and POPDC is independent of cAMP production, while AC9 association with TREK‐1 is reduced in an isoproterenol (ISO)‐dependent manner, requiring an intact Popeye domain and local production of cAMP within the complex. Moreover, AC9 regulates POPDC1 effects on TREK‐1 currents. POPDC1 therefore represents a novel adaptor protein for AC9 to regulate downstream effectors for HR control.

## Results

### Deletion of *Adcy9* decreases HR at rest and increases HR variability after ISO injection

**Figure 1:**
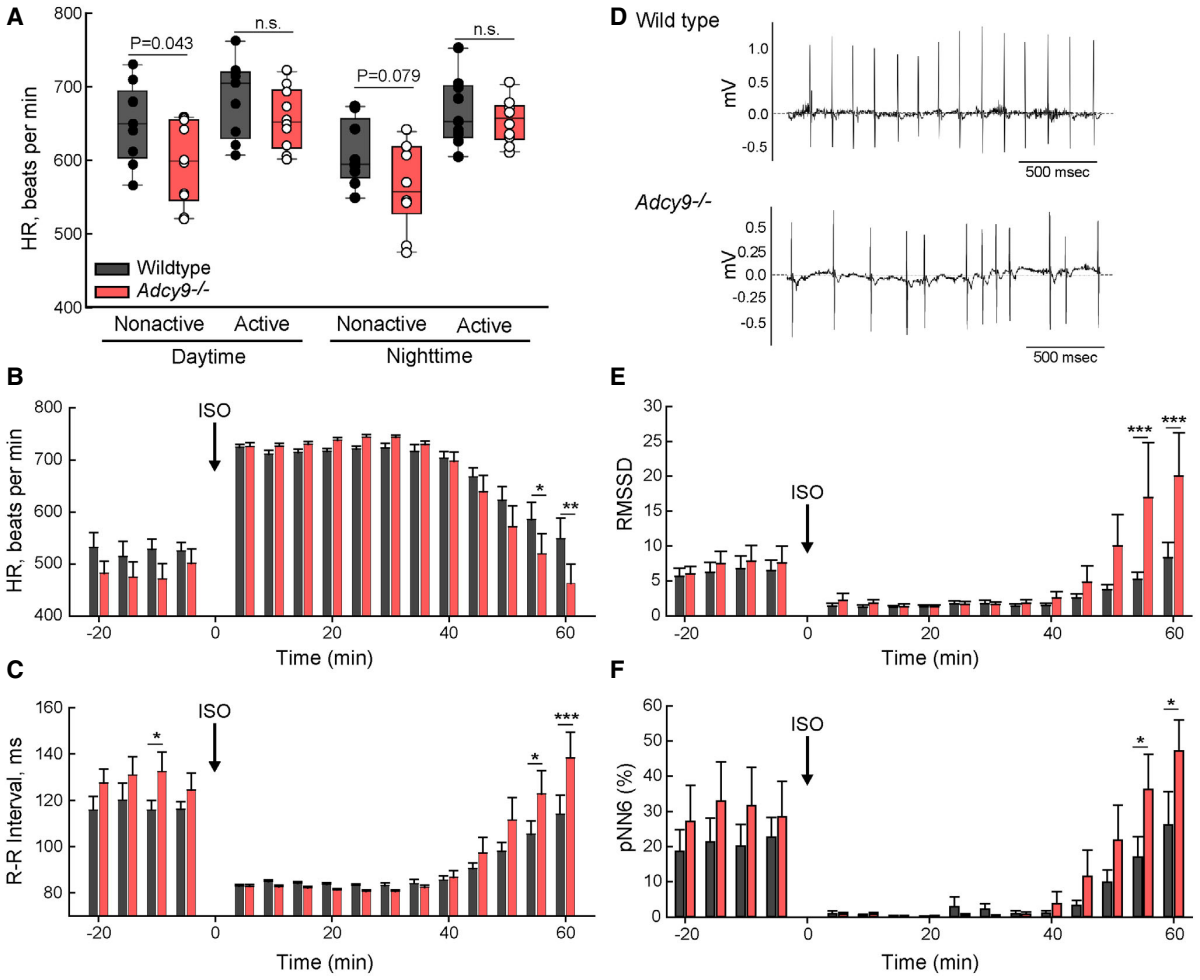
Deletion of *Adcy9* results in bradycardia at rest and HR variability during the recovery period after ISO injection A Heart rate (HR) of WT and *Adcy9* ^−/−^ mice averaged over 5 min of nonactive versus sustained activity on home cage running wheels. The Student's *t*‐test *P*‐values for indicated comparisons are shown. Circles represent data from individual animals. The boxplot central band is the median while whiskers represent the 10^th^ and 90^th^ percentiles.B, CHR and R‐R interval of WT (black bars) and *Adcy9* ^−/−^ mice (red bars) before and after ISO (1 μg/g) injection; mean of 5 min intervals ± SE are shown.DRepresentative ECG recordings of 7 months WT and *Adcy9* ^−/−^ mice, ~ 55 min post‐ISO injection.E, FHR variability was calculated before and after ISO injection using the root mean square of successive RR interval differences (RMSSD; panel (E)) and percentage of sequential R‐R intervals differing by >6 ms (pNN6; panel (F)) methods. Data information: Two‐way ANOVA repeated‐measures analysis (time and genotype) with the Holm–Sidak method used for pairwise comparisons for HR, RR, and HR variability with ISO; *n* = 9 WT and *n* = 10 *Adcy9* ^−/−^ mice; *P < 0.05; **P < 0.01; ***P < 0.001. Source data are available online for this figure.

Previous studies using Doppler imaging of isoflurane‐anesthetized mice revealed a mild bradycardia in *Adcy9* ^−/−^ male and female mice from 1 to 7 months of age (Li *et al*, 2017). To confirm the effects of *Adcy9* deletion on HR in conscious animals, we measured electrocardiograms (ECGs) by telemetry. HR was measured over a 24‐h period in which the animals were given free access to a running wheel. *Adcy9* ^−/−^ mice displayed a daytime bradycardia during periods of rest (defined as no movement of the running wheel for > 10 min), but not while actively moving (> 5 min on running wheel; Fig 1A). Similar trends were also observed at night.

Mice were subjected to behavioral tests postimplantation of telemetry devices to rule out effects on general locomotor activity levels and exploratory drive (running wheels and open field activity box) or anxiety (elevated plus maze and forced swim test) that may affect overall HR. Overall, no significant differences were found in the maximal velocities or total distance traveled on the running wheel or during the 25 min open field test (Table 1). *Adcy9* ^−/−^ also showed no exploratory differences in the open field test and displayed similar levels of anxiety and depression‐like behavior as demonstrated in the elevated plus maze and forced swim tests (Table 1).

**Table 1:** Behavioral assays for WT and *Adcy9* ^−/−^ mice.

Tests^a^	WT	*Adcy9* ^−/−^	*P*‐value*
Running wheel			
Distance, 7 pm – 7 am (m)	1,700 ± 1,080	1,070 ± 790	0.20
Distance, 1^st^ h (m)	180 ± 120	140 ± 90	0.48
Max velocity (m/min)	25 ± 15	24 ± 22	0.92
Open field box			
Zone 1 (cm)	301 ± 126	431 ± 199	0.32
Zone 1 entries	66 ± 39	62 ± 27	0.50
Total distance	1,701 ± 350	2,250 ± 1,170	0.48
Avg velocity (cm/min)	43.3 ± 11.1	40.7 ± 12.0	0.65
EPM			
Time, open arm (s)	24 ± 13	18 ± 24	0.25
Forced swim			
Immobility (s)	147 ± 36	158 ± 41	0.58

^a^ Mean ± SD is given for combined male and female animals.

* Student's *t*‐tests were performed, except for EPM, which was analyzed by the Mann–Whitney rank‐sum test, *n* = 9 WT mice (4 females, 5 males), *n* = 10 *Adcy9* ^−/−^ mice (4 females, 6 males). Source data are available online for this table.

To induce an increase in HR in a controlled and timed manner, we administered ISO (1 μg/g) by a single intraperitoneal injection and recorded ECGs. Both genotypes and sexes showed a substantial increase in HR upon ISO administration (39 ± 10% and 48 ± 23% for WT and *Adcy9* ^−/−^, respectively; Fig 1B), reaching a similar maximal HR. However, *Adcy9* ^−/−^ displayed a more rapid recovery to baseline with significantly longer R‐R intervals (the time between two successive R‐waves of the ECG) and slower HR (the reciprocal of R‐R) at 55 and 60 min postinjection (Fig 1B and C). A further examination of ECG traces revealed a variability in beat‐to‐beat timing in *Adcy9* ^−/−^ mice at these time points (Fig 1D). HR variability was quantified by two distinct methods, which calculate the beat‐to‐beat consistency: RMSSD (Root mean square of successive RR interval differences) and pNN (Percentage of successive RR intervals that differ by more than 6 ms). Both methods showed statistically significant HR variability for *Adcy9* ^−/−^ mice in the recovery phase after ISO injection (Fig 1E and F).

### POPDC proteins bind AC9 in HEK293 cells and cardiomyocytes

Despite the effects on HR and HR variability during recovery from beta‐adrenergic stimulation, it was not immediately clear why deletion of *Adcy9* would give rise to this phenotype. AC9 is present in the sinoatrial (SA) node (Li *et al*, 2017), but the Ca^2+^/calmodulin‐stimulated ACs are generally thought to be responsible for pacemaker activity within the SA node (Mattick *et al*, 2007; Kryukova *et al*, 2012; Moen *et al*, 2019; Robinson *et al*, 2020). Thus, we searched the literature for proteins that were regulated by cAMP and when deleted, displayed HR variability. Two proteins of interest included POPDC1 and TREK‐1; upon deletion in mice, both mutants show a sinoatrial pacemaking phenotype and HR variability (Froese *et al*, 2012; Unudurthi *et al*, 2016). POPDC proteins represent a family of cAMP effectors that bind to the two‐pore potassium channel TREK‐1 (Brand, 2019; Swan *et al*, 2019). Additionally, a high‐throughput affinity purification‐mass spectrometry screen identified AC9 and AC3 with POPDC2 as bait; however, these potential interactions were never biochemically verified (Huttlin *et al*, 2015).

**Figure EV1:**
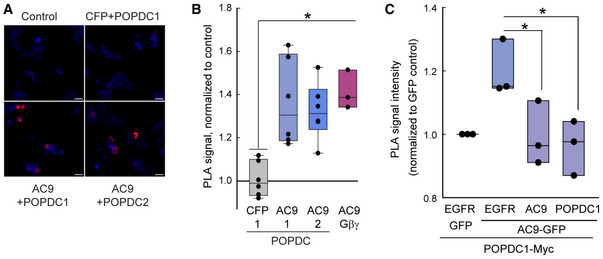
PLA signal between GFP‐tagged AC9 and POPDC1‐Myc A, BImages (A) of PLA signal (red) and DAPI (blue) performed in HEK293 cells using antibodies against GFP and MYC tags. Scale bars are 20 μm. Mean cellular fluorescence intensity of PLA signal (B) was quantified by high content microscopy. The Kruskal–Wallis one‐way ANOVA analysis was performed (*n* = 7 experiments, *P* = 0.003 between groups) with multiple comparisons by the Bonferroni *t*‐test (*P < 0.05). Boxplots show the median as the central band, the box size as the lower and upper quartiles, while the 10^th^ and 90^th^ percentiles are represented by the whiskers.CCompetition of PLA signal between GFP‐tagged AC9 and POPDC1‐Myc with overexpression of nontagged EGFR (control), Flag‐AC9, or POPDC1‐Flag in HEK293 cells. Mean cellular fluorescence intensity was quantified by high content microscopy and normalized to GFP control. The Kruskal–Wallis one‐way ANOVA analysis was performed (*n* = 3 experiments, >1,000 cells per experiment) with multiple comparisons by the Student–Newman–Keuls method (*P < 0.05). Boxplots show the median as the central band, the box size as the lower and upper quartiles, while the whiskers are the range.

Unfortunately, AC9 and POPDC1 suffer from a lack of antibodies sufficient for immunoprecipitation, with poor detection of endogenous proteins by western blotting. Therefore, we first tested for potential interactions between AC9 and POPDC using the expression of tagged proteins in cellular assays of HEK293 cells and COS‐7 cells. These tagged proteins are fully functional, as demonstrated by activity assays and AKAP association for AC9 (Li *et al*, 2012, 2017; Baldwin *et al*, 2019; Lazar *et al*, 2020) and cAMP binding and TREK‐1 interactions for POPDC1 (Froese *et al*, 2012; Schindler *et al*, 2016b). Proximity ligation assay (PLA) can amplify the detection of protein–protein interactions that occur within a range of < 60 nm (Fredriksson *et al*, 2002). YFP‐tagged AC9 and POPDC‐MYC‐tagged proteins were expressed in HEK293 cells; interactions between AC9 and Gβγ (as detected with antibodies against Gβ) were used as a positive control (Li *et al*, 2017). PLA puncta (red) are readily detected with AC9 and POPDC isoforms 1 and 2, suggesting close proximity and possible complex formation (Fig EV1A and B). As a control, overexpression of nontagged POPDC1 or AC9 was used to compete with the PLA signal between tagged POPDC1 and AC9 (Fig EV1C). These nontagged proteins reduced the PLA signal compared with the noninteracting transmembrane protein EGFR.

**Figure 2:**
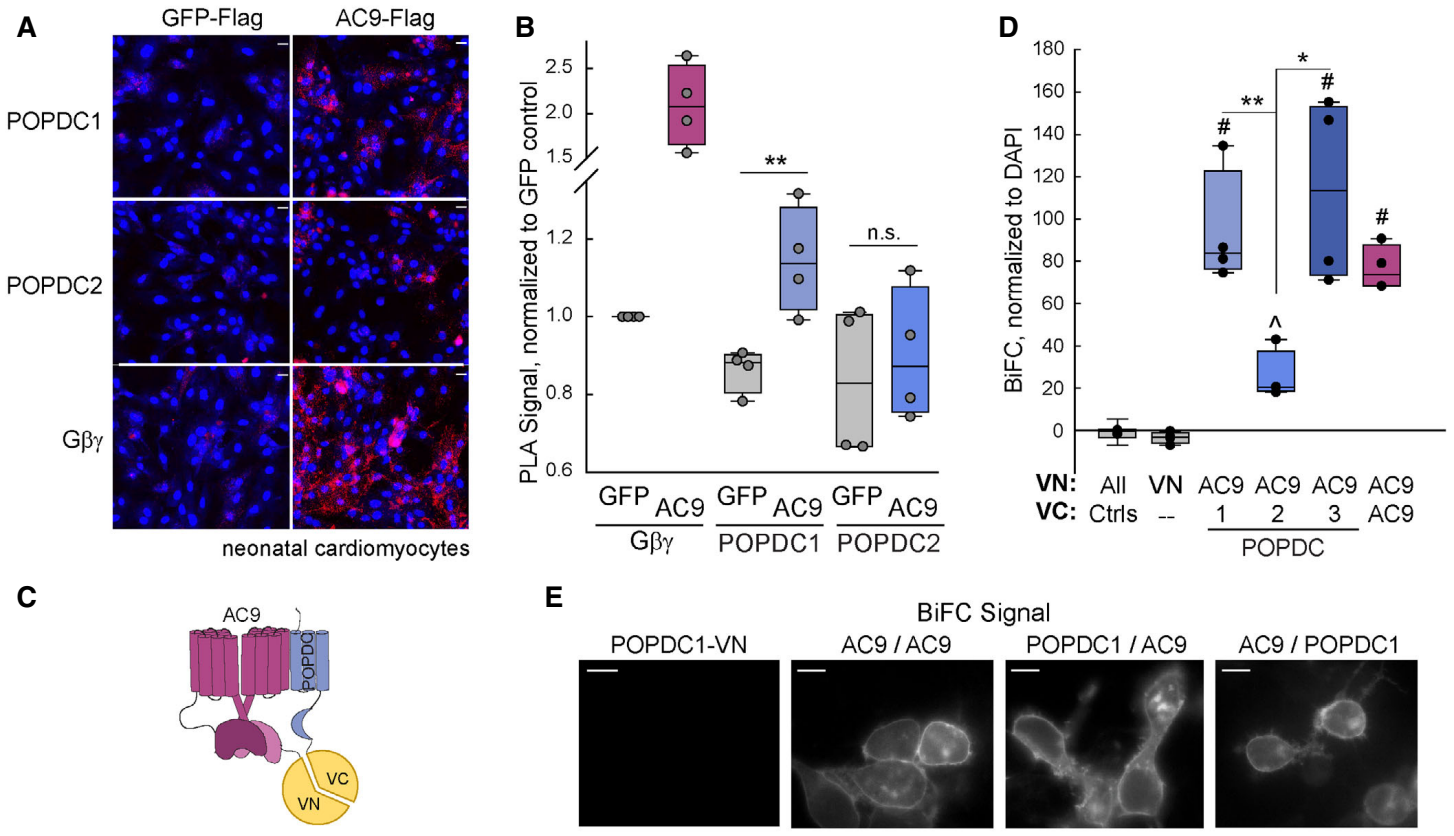
Cellular interaction of POPDC isoforms with AC9 A, BPLA assay performed in neonatal cardiomyocytes expressing GFP‐Flag or AC9‐Flag using antibodies against Flag and endogenous POPDC1, POPDC2, or Gβγ. Images (A); scale bar is 20 μm; nuclear staining by DAPI shown in blue) and quantitation (B) of PLA signal is shown. The unpaired two‐sided Student's *t*‐test (*n* = 4 independent experiments, **P = 0.0086) was performed for each antibody pair. Boxplots show the median as the central band, the box size as the lower and upper quartiles, while the whiskers are the range.CCartoon of AC9‐POPDC BiFC complex.DQuantification of COS‐7 cells expressing BiFC constructs for AC9, POPDC 1–3. The expressed proteins tagged with VN (top line) and VC (bottom line) are shown. The Kruskal–Wallis one‐way ANOVA analysis on ranks was performed (*n* = 4 experiments) with multiple comparisons with control by the Dunnett's method (^#^ *P* < 0.05). Individual comparisons were performed with a two‐tailed Student's *t*‐test (**P = 0.003, *P = 0.018) or Mann–Whitney rank‐sum test (^*P* = 0.004 versus control) Boxplots show the median as the central band, the box size as the lower and upper quartiles, while the whiskers are the range.ERepresentative live‐cell images of indicated BiFC combinations in HEK293 cells (*n* > 20 cells; scale bar: 10 μm). Quantification of POPDC1‐VN:AC9‐VC is shown in Figs 3 and 7. Source data are available online for this figure.

AC9 also interacts with endogenous POPDC1 in neonatal cardiomyocytes (CMs). Using antibodies that were validated for immunohistochemistry against endogenous POPDC1 or POPDC2 (see methods), PLA signals were detected in CMs between POPDC1 and adenovirally expressed Flag‐tagged AC9 but not Flag‐tagged GFP (Fig 2A and B). Interaction of AC9 with endogenous Gβγ served as a positive control, while PLA signals were not significant for endogenous POPDC2 and AC9. A complementary cellular interaction technique is Bimolecular Fluorescence Complementation (BiFC). Here, potential interacting pairs were tagged with either the N‐terminal half of Venus (VN) or C‐terminal half of Venus (VC; Fig 2C). Since HEK293 cells express endogenous POPDC1 and POPDC2, we performed BiFC experiments in COS‐7 cells that lack detectable endogenous POPDC proteins (Appendix Table S1). A strong BiFC signal was detected between AC9 and POPDC 1 and 3, and to a lesser extent with POPDC2 (Fig 2D), with AC9:POPDC1 BiFC signal localizing to PM in HEK293 cells (Fig 2E).

**Figure 3:**
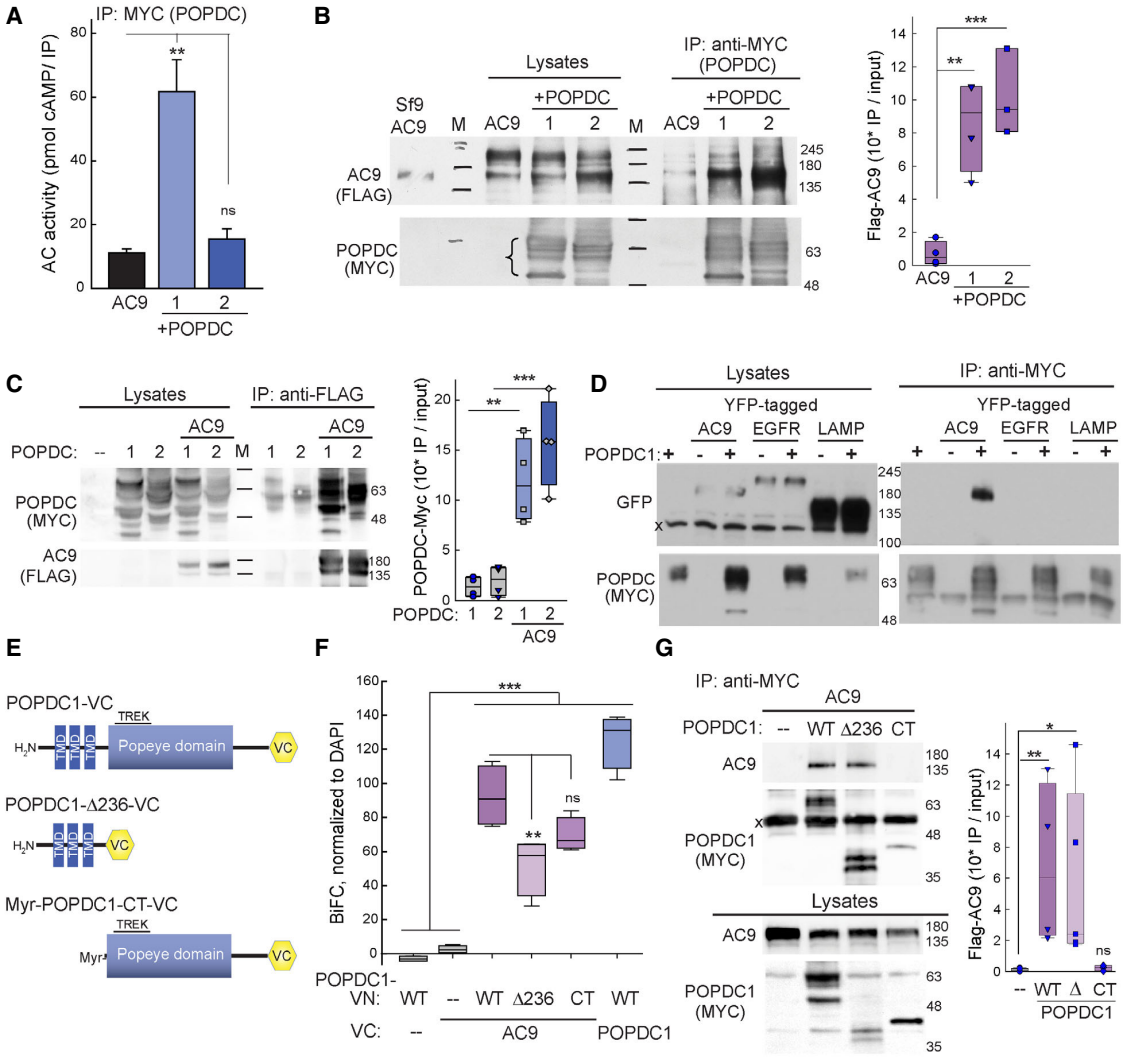
AC9 interacts with the POPDC1 transmembrane and Popeye domains HEK293 cells expressing Flag‐AC9 in the presence or absence of Myc‐tagged POPDC1 or − 2 were subjected to co‐immunoprecipitation (Co‐IP) with anti‐MYC and assayed for AC activity with 300 nM Gαs‐GTPγS. The Kruskal–Wallis one‐way ANOVA analysis on ranks was performed (*n* = 6 experiments, *P* = 0.003 between all groups) with multiple comparisons with AC9 control by the Dunn's method and Mann–Whitney rank‐sum test (**P = 0.002). Data are plotted as mean ± SEM.A portion of the lysates and Co‐IP from (A) were subjected to western blot (WB) analysis. Sf9 cells expressing Flag‐AC9 served as a positive WB control. Molecular weight markers are denoted as M. Quantitation of Flag‐AC9 WB by one‐way ANOVA, *n* = 3–4 experiments, with comparisons by the Tukey test, **P = 0.003, ***P = 0.001. Boxplots show the median as the central band, the box size as the lower and upper quartiles, while the whiskers are the range.HEK293 cells expressing Flag‐AC9 +/− POPDC1‐Myc or POPDC2‐Myc were subjected to Co‐IP with anti‐FLAG and subjected to WB analysis with anti‐MYC and anti‐FLAG. Quantitation of anti‐MYC WB by one‐way ANOVA, *n* = 4 experiments, with comparisons by the Tukey test, **P = 0.003, ***P < 0.001). Boxplots show the median as the central band, the box size as the lower and upper quartiles, while the whiskers are the range.HEK293 cells expressing POPDC1‐Myc +/− GFP‐tagged AC9 and control TM proteins EGFR, or LAMP1 were subjected to Co‐IP with anti‐MYC. Western blotting of lysates and Co‐IPs for GFP (top) and MYC (bottom) are shown (*n* = 3 experiments).Schematic of POPDC1 truncations.BiFC of AC9 and indicated POPDC1 truncations in COS‐7 cells. The Kruskal–Wallis one‐way ANOVA analysis was performed (*n* = 4 experiments) with multiple comparisons by the Tukey test, ***P < 0.001 compared with VN control, **P = 0.008. Boxplots show the median as the central band, the box size as the lower and upper quartiles, while the whiskers are the range.Co‐IP with anti‐MYC in COS‐7 cells expressing Flag‐AC9 and indicated Myc‐tagged POPDC1 truncations. Western blotting with anti‐AC9 and anti‐MYC (POPDC1) of Co‐IP and lysates is shown. The Kruskal–Wallis one‐way ANOVA analysis was performed (*n* = 3–5 experiments, *P* = 0.006), with multiple comparisons by the Dunn's method (*P = 0.021, **P = 0.01). Boxplots show the median as the central band, the box size as the lower and upper quartiles, while the whiskers are the range. Source data are available online for this figure.

To test whether AC and POPDC interactions could also be detected by co‐immunoprecipitation (co‐IP), Flag‐tagged AC9 was co‐expressed with or without Myc‐tagged POPDC1 or POPDC2 in HEK293 cells. Complexes were isolated with anti‐MYC antibodies and associated Gαs‐stimulated AC activity was measured (IP‐AC assay; Fig 3A). From the IP‐AC assay, it appeared that AC9 interacted with POPDC1 but not POPDC2. However, when the co‐IPs were evaluated via western blot, AC9 co‐precipitates with both POPDC1 and POPDC2 (Fig 3B). Thus, POPDC1 pulls down AC9 and maintains associated AC activity, while AC9 complexed with POPDC2 does not respond to Gαs stimulation. POPDC2:AC9 may represent a different conformation than POPDC1:AC9, consistent with the decreased BiFC and PLA signals and AC9‐associated activity. POPDC interactions with AC9 were confirmed by performing co‐IP in reverse by pulling down the complex with the associated Flag‐tag on AC9 (Fig 3C). POPDC1 and POPDC2 were both detected in pull‐downs of Flag‐AC9.

A number of POPDC interacting proteins have been identified to date, many of which are transmembrane proteins (reviewed in Schindler & Brand, 2016). To verify the specificity of our immunoprecipitation conditions, we confirmed the association of POPDC1 with AC9 but not the transmembrane epidermal growth factor receptor (EGFR) or lysosomal associated membrane protein 1 (LAMP1; Fig 3D). To begin to narrow down the site of AC9:POPDC1 interaction, the transmembrane domain (POPDC1Δ236) or the PM‐targeted cytosolic Popeye domain (Myr‐POPDC1‐CT) were tested for interactions with AC9 (Fig 3E–G). Both the transmembrane and cytosolic domain of POPDC1 interacted with AC9 by BiFC, although deletion of the cytosolic domain gave rise to a reduced BiFC signal compared with the full‐length protein. This may be due to alterations in the distance between VN and VC proteins with the truncated POPDC1, as POPDC1Δ236 interacted with AC9 by co‐IP, similar to full‐length POPDC1. Interactions between AC9 and the cytosolic domain of POPDC1 were not observed by pull‐down, suggesting that although both domains participate in interactions with AC9. The stronger interaction likely occurs within the transmembrane‐spanning regions.

### POPDC1 mediates interaction of TREK‐1 with AC9

**Figure 4:**
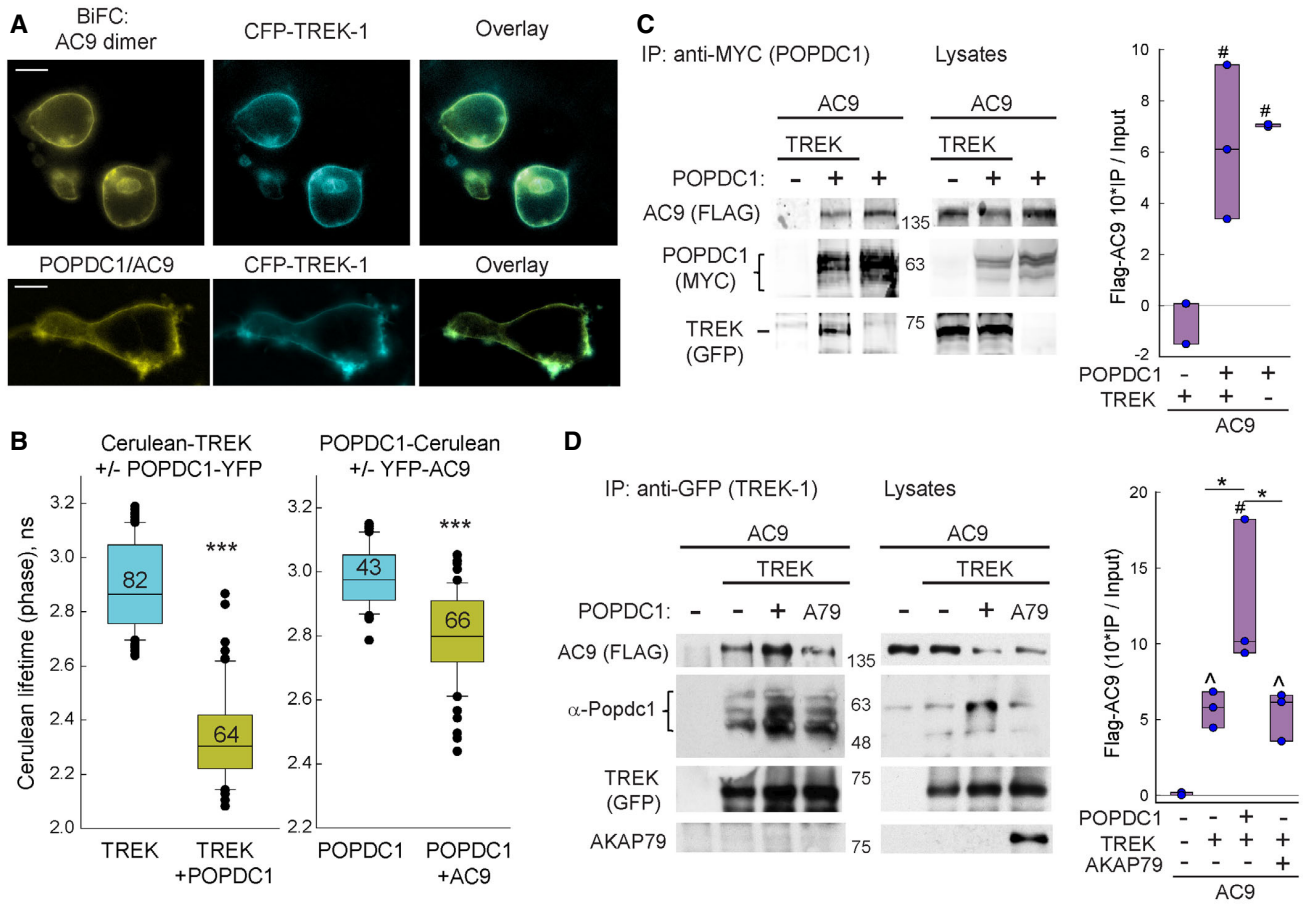
AC9 is part of a POPDC1‐TREK‐1 complex Co‐localization of CFP‐TREK‐1 with the BiFC signal from AC9‐VN:AC9‐VC homodimer (top) and POPDC1‐VN:AC9‐VC complex (bottom) in HEK293 cells. Scale bar: 10 μm.The lifetime distribution of Cerulean‐tagged proteins +/− the indicated YFP‐tagged proteins expressed in HEK293 cells. The Mann–Whitney rank‐sum test was performed (***P < 0.001; *n* = cell number indicated on each bar). Boxplots show the median as the central band, the box size as the lower and upper quartiles, while the whiskers are the range.Co‐IP of Myc‐tagged POPDC1 (anti‐MYC) pulled down both Flag‐AC9 and YFP‐tagged TREK‐1 (*n* = 3 experiments). The one‐way ANOVA was performed with multiple comparisons by the Holm–Sidak method (^#^ *P* = 0.007 compared with control). Boxplots show the median as the central band, the box size as the lower and upper quartiles, while the whiskers are the range.Co‐IP of TREK‐1 (anti‐GFP) pulls down Flag‐AC9 and endogenous POPDC1. AKAP79 is not pulled down in complex (*n* = 3 experiments). The one‐way ANOVA was performed with multiple comparisons by the Student–Newman–Keuls method (^#^ *P* < 0.05 compared with control; *P = 0.013 and 0.026 comparing bars 2–3 and 3–4, respectively) or two‐tailed Welch's *t*‐test for nonequal variances (^*P* = 0.014 and ^*P* = 0.029). Boxplots show the median as the central band, the box size as the lower and upper quartiles, while the whiskers are the range. Data information: Quantification of Flag‐AC9 WB (IP/total expression in lysate) is shown to the right of (C) and (D). Source data are available online for this figure.

As POPDC1 interacts with TREK‐1 (Froese *et al*, 2012), we sought to determine whether AC9 could associate with a POPDC1:TREK‐1 complex. TREK‐1 co‐localized in HEK293 cells with the BiFC signal from the AC9 homodimer (Fig 4A, top) and the BiFC complex of POPDC1:AC9 (Fig 4A, bottom), suggesting that all three proteins co‐localize on the plasma membrane. Cellular interactions can be observed by fluorescence lifetime microscopy (FLIM) of Cerulean‐tagged proteins to quantitatively measure FRET efficiency with YFP‐tagged partners (Fig 4B). Using this method, interactions were observed between TREK‐1 and POPDC1 (18 ± 2% FRET efficiency) and POPDC1 and AC9 (6.2 ± 0.4% FRET). The low FRET efficiency is likely due to the distance constraints of FRET, given that all three proteins (TREK‐1, POPDC1, and AC9) form dimers (Kawaguchi *et al*, 2008; Lolicato *et al*, 2017; Baldwin *et al*, 2019). Despite the low FRET efficiency, immunoprecipitation of POPDC1 pulled down both AC9 and TREK‐1 when co‐expressed in HEK293 cells (Fig 4C). Similarly, immunoprecipitation of YFP‐tagged TREK‐1, but not YFP alone, pulled down AC9 and endogenous or overexpressed POPDC1 (Fig 4D; Appendix Fig S1). TREK‐1 also interacts with AKAP79 (Sandoz *et al*, 2006). However, overexpression of AKAP79 did not enhance AC9 pull‐down with TREK‐1, nor was AKAP79 detected in immunoprecipitates of TREK‐1 (Fig 4D).

### Association of TREK‐1 with AC9 activity in heart requires POPDC1 expression

**Figure 5:**
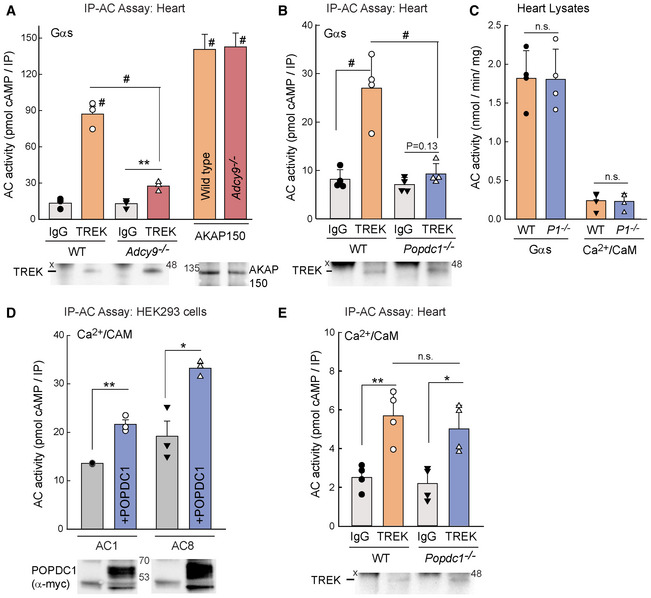
TREK‐1 pulls down AC9 activity in heart, which requires POPDC1 expression IP‐AC assay with IgG versus anti‐TREK‐1 in heart homogenates from WT and *Adcy9* ^−/−^ mice; AC activity stimulated with 300 nM Gαs‐GTPγS. IP‐AC with anti‐AKAP150 is shown as a positive control. The two‐way ANOVA (overall *P* < 0.001, *n* = 3 mice per genotype) with multiple comparisons by the Holm–Sidak test; ^#^ *P* < 0.001 for indicated comparison or to respective IgG controls. IgG versus anti‐TREK‐1 in *Adcy9* ^−/−^ was analyzed by the Student's *t*‐test (**P = 0.009, *n* = 3). A portion of the co‐IP was subjected to western blotting with anti‐TREK‐1 and anti‐AKAP150. Data are plotted as mean ± SEM.IP‐AC assay with IgG versus anti‐TREK in WT and *Popdc1* ^−/−^ mouse heart homogenates; AC activity stimulated as in (A). Statistics as in (A), *n* = 4 mice per genotype, #*P* < 0.001. Paired *t*‐test within *Popdc1* ^−/−^, *P* = 0.123. Data are plotted as mean ± SD.Total AC activity in heart homogenates from WT or *Popdc1* ^−/−^ (*P1* ^−/−^) mice stimulated with 300 nM Gαs‐GTPγS or 100 μM calcium and 300 nM calmodulin. Student's *t*‐test (*n* = 4, n.s.). Data are plotted as mean ± SD.IP‐AC assay with anti‐MYC from cell lysates expressing AC1 or AC8 in the presence or absence of Myc‐tagged POPDC1. AC activity stimulated with 100 μM calcium and 300 nM calmodulin. Student's *t*‐test (*n* = 3 experiments, **P = 0.0011 and *P = 0.012). Data are plotted as mean ± SEM.IP‐AC assay with IgG versus anti‐TREK‐1 in WT and *Popdc1* ^−/−^ mouse heart homogenates; AC activity stimulated as in (D). Student's *t*‐test (*P = 0.03; **P = 0.005, *n* = 4 mice per genotype). Data are plotted as mean ± SD. Source data are available online for this figure.

To further validate the interaction between TREK‐1 and AC9 in cardiac tissue, we measured the AC activity that was associated with immunoprecipitates of TREK‐1 in hearts from WT or *Adcy9* ^−/−^ mice. The TREK‐1 antibody was validated for immunoprecipitation studies using YFP‐tagged TREK‐1 expression (Appendix Fig S2). TREK‐1 antibodies pulled down 8 ± 2‐fold more Gαs‐stimulated AC activity than IgG controls in heart from WT mice, while AC activity associated with TREK‐1 pull‐down was significantly reduced, but not eliminated, in *Adcy9* ^−/−^ mice (2.2 ± 0.6‐fold compared with IgG controls; Fig 5A). Note that in WT and *Adcy9* ^−/−^ mice, the total bulk AC activity and AKAP150‐associated AC activity was unchanged (Fig 5A; Li *et al*, 2017). POPDC1 was required for the association of Gαs‐stimulated AC activity with TREK‐1, as deletion of *Popdc1* nearly eliminated all TREK‐1 associated AC activity (Fig 5B). However, this was not due to alterations of total AC activity in *Popdc1* ^−/−^ hearts (Fig 5C).

### POPDC1 expression is not required for TREK‐1 association with Ca^2+^/calmodulin‐stimulated AC activity

POPDC1 may interact with multiple AC isoforms, accounting for the remaining TREK‐1‐associated AC activity in immunoprecipitations from *Adcy9* ^−/−^ mice (Fig 5A). The calcium‐activated AC isoforms, AC1 and AC8, present in atria and SA node, regulate pacemaker activity in the SA node (Robinson *et al*, 2020). To determine whether AC1 or AC8 interacts with POPDC1, nontagged AC1 and AC8 were expressed with Myc‐tagged POPDC1 in HEK293 cells and an IP‐AC assay was performed. POPDC1 is weakly associated with AC1 and AC8 Ca^2+^/calmodulin‐stimulated AC activity (1.59 ± 0.05 and 1.8 ± 0.2 fold over IgG controls, respectively; Fig 5D), demonstrating that multiple cardiac AC isoforms can potentially complex with POPDC1. In cardiac tissue, TREK‐1 also pulled down Ca^2+^/calmodulin‐stimulated AC activity (2.8 ± 1.3 fold over IgG controls), suggesting that AC1 and/or AC8 may participate in the regulation of TREK‐1 (Fig 5E). However, deletion of POPDC1 did not alter the amount of Ca^2+^/calmodulin‐stimulated AC activity associated with TREK‐1 in the heart (Fig 5C). Therefore, POPDC1 is required for the association of Gαs‐stimulated, but not Ca^2+^/calmodulin‐stimulated, AC activity with TREK‐1.

### AC9 interacts with TREK‐1 in an ISO‐dependent manner that requires POPDC1

**Figure 6:**
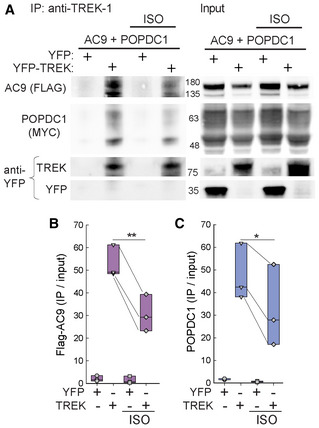
Isoproterenol treatment reduces AC9 and POPDC1 interaction with TREK‐1 ATreatment of HEK293 cells for 10 min in the presence of 10 μM ISO reduced the amount of Flag‐AC9 and POPDC1‐Myc pulled down by TREK‐1 co‐IP.B, CQuantification (IP/input) of Flag‐AC9 (B) and POPDC1‐Myc (C) between ISO treatment groups by a two‐tailed paired Student's *t*‐test (*P = 0.028, **P = 0.007; *n* = 3 biological replicates). Boxplots show the median as the central band, the box size as the lower and upper quartiles, while the whiskers are the range. Source data are available online for this figure.

Cyclic AMP binding to POPDC proteins decreases TREK‐1:POPDC interactions (Froese *et al*, 2012). To determine whether AC9 interactions with TREK‐1 were also dependent on cAMP, we examined the effect of ISO on complex formation between AC9, POPDC1, and TREK‐1. HEK293 cells expressing YFP‐tagged TREK‐1, Flag‐AC9, and POPDC1‐Myc were treated with ISO for 10 min prior to co‐IP with TREK‐1 antibodies (Fig 6A). Treatment with ISO reduced the amount of AC9 and POPDC1 associated with TREK‐1 (Fig 6B and C).

**Figure 7:**
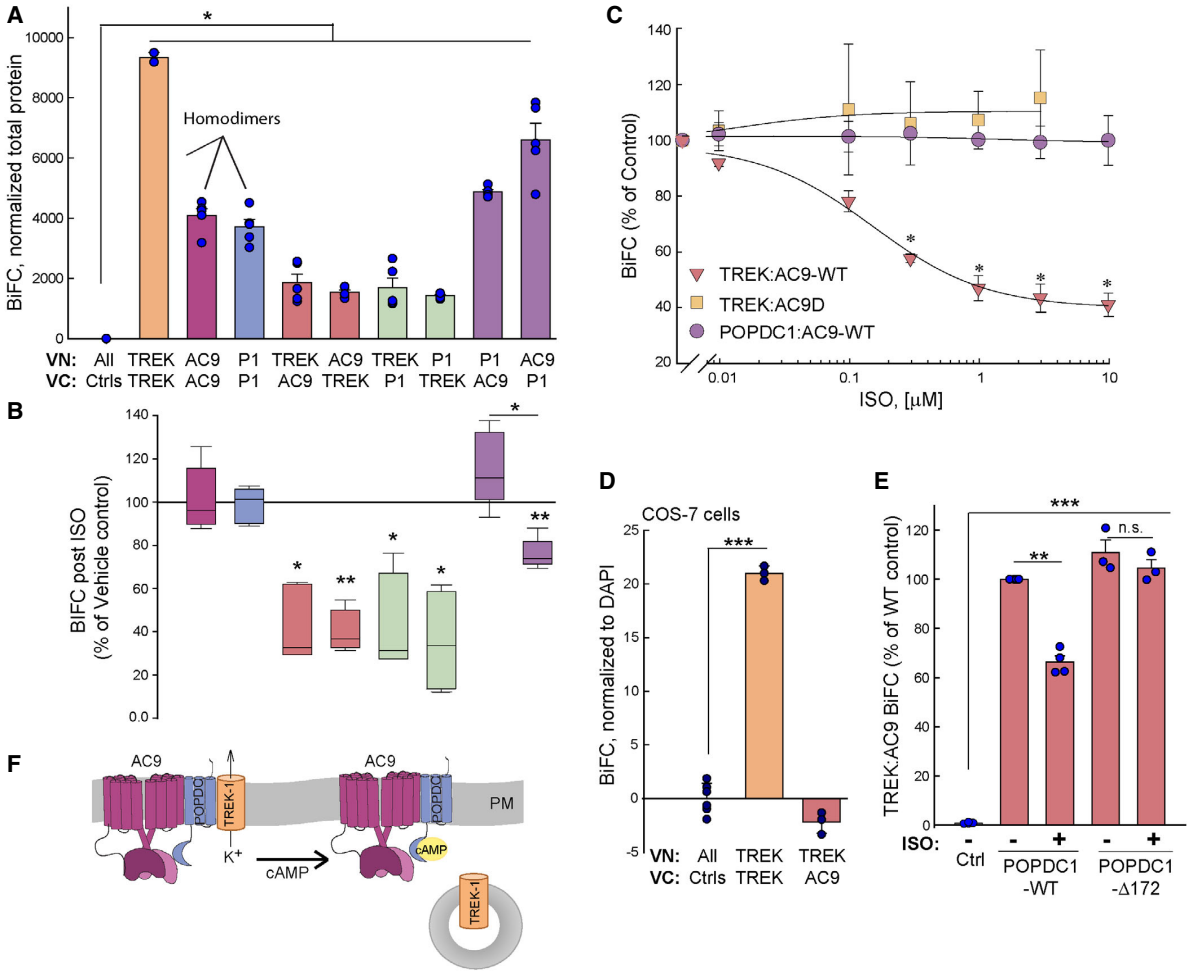
ISO‐dependent loss of TREK‐1 from AC9‐POPDC1 complex requires an intact Popeye domain ABiFC signal between the indicated VN‐ and VC‐tagged proteins (POPDC1, P1). Kruskal–Wallis one‐way ANOVA on ranks (*n* = 5 experiments, *P* < 0.001 between groups) with multiple comparisons with VN and VC controls by Student–Newman–Keuls tests (*P < 0.05). Data are plotted as mean ± SEM.BPercent decrease of BiFC signal in (A) with ISO treatment (10 μM, 10 min at 37°C). Paired *t*‐test for each condition (vehicle versus ISO) using raw data prior to calculation of percent of vehicle control (*n* = 6; *P < 0.05; **P < 0.01). Boxplots show the median as the central band, the box size as the lower and upper quartiles, while the whiskers are the range.CISO dose–response curves for BiFC interactions between POPDC1:AC9, TREK‐1:AC9, and TREK‐1 with a catalytically inactive AC9 (TREK‐1:AC9D). Two‐way ANOVA with multiple comparisons by the Bonferroni method (*n* = 3–4; *P < 0.05 for comparisons between TREK:AC9 versus TREK:AC9D and POPDC1:AC9). Data are plotted as mean ± SEM.DTREK‐1:AC9 BiFC signal is absent in COS‐7 cells lacking detectable POPDC1. TREK‐1:TREK‐1 BiFC is a positive control. One‐way ANOVA with multiple comparisons by the Holm–Sidak method (*n* = 3 experiments; ***P < 0.001). Data are plotted as mean ± SD.EOverexpression of POPDC1 truncation of Popeye domain (POPDC1‐Δ172), but not WT POPDC1, abolishes ISO reduction in TREK‐1:AC9 BiFC signal. One‐way ANOVA with multiple comparisons by the Holm–Sidak method (*n* = 3–4 experiments; ***P < 0.001 compared with control). Data are plotted as mean ± SEM.FModel of cAMP effects on AC9‐POPDC1‐TREK‐1 complex formation. Source data are available online for this figure.

To further probe the regulation of the TREK‐1:POPDC1:AC9 complex by beta‐adrenergic stimulation, BiFC was employed to probe all possible interactions. BiFC signals were generally lower for the pairs, TREK‐1:AC9 or TREK‐1:POPDC, as compared to AC9:POPDC1 or dimer formation by TREK‐1, AC9, or POPDC1 (Fig 7A). Upon ISO treatment (10 min, 10 μM), the overall BiFC signal for TREK‐1:AC9 or TREK‐1:POPDC1 was decreased approximately 50–65%, independent of which protein was tagged with the amino or carboxy‐terminal half of Venus (Fig 7B). ISO treatment did not alter VN‐ or VC‐tagged protein levels (Appendix Fig S3). The IC_50_ for the ISO‐induced decrease in TREK‐1:AC9 BiFC signal was 160 ± 40 nM (Fig 7C). ISO regulation of TREK‐1 required both an active AC9 enzyme and endogenous expression of POPDC1 (Fig 7C and D). TREK‐1 interactions with a catalytically dead AC9 were not sensitive to ISO, suggesting that local cAMP production was important (Fig 7C). Endogenous expression of POPDC1 was also required for ISO regulation of TREK‐1 interactions, as no TREK‐1:AC9 BiFC signal was observed in COS‐7 cells, which lack detectable POPDC1 (Fig 7D; Appendix Table S1). Finally, an intact Popeye domain was required, as overexpression of a truncated POPDC1 protein (POPDC1Δ172), which lacks cAMP binding but retains TREK‐1 association (Schindler *et al*, 2016b), acted as a dominant negative to block the effects of ISO (Fig 7E). Expression of WT POPDC1 had no effect on TREK‐1:AC9 BiFC signals. The interaction between AC9 and POPDC1 showed either no change or a 24 ± 3% decrease with ISO, depending on which protein was tagged with VN and VC (Fig 7B). This dependence on VN/VC tagging may represent a change in orientation between the two proteins upon either Gαs interaction with AC9 or POPDC1 binding of cAMP. Neither AC9 nor POPDC1 homodimers showed alterations in BiFC responses with ISO treatment (Fig 7B). These data support a model where local cAMP production by AC9 enhances POPDC1 binding of cAMP to drive the breakdown of the AC9:POPDC:TREK‐1 assembly, releasing TREK‐1 from the more stable AC9:POPDC1 complex (Fig 7F).

### AC9 activity opposes POPDC1 enhancement of TREK‐1 currents

**Figure 8:**
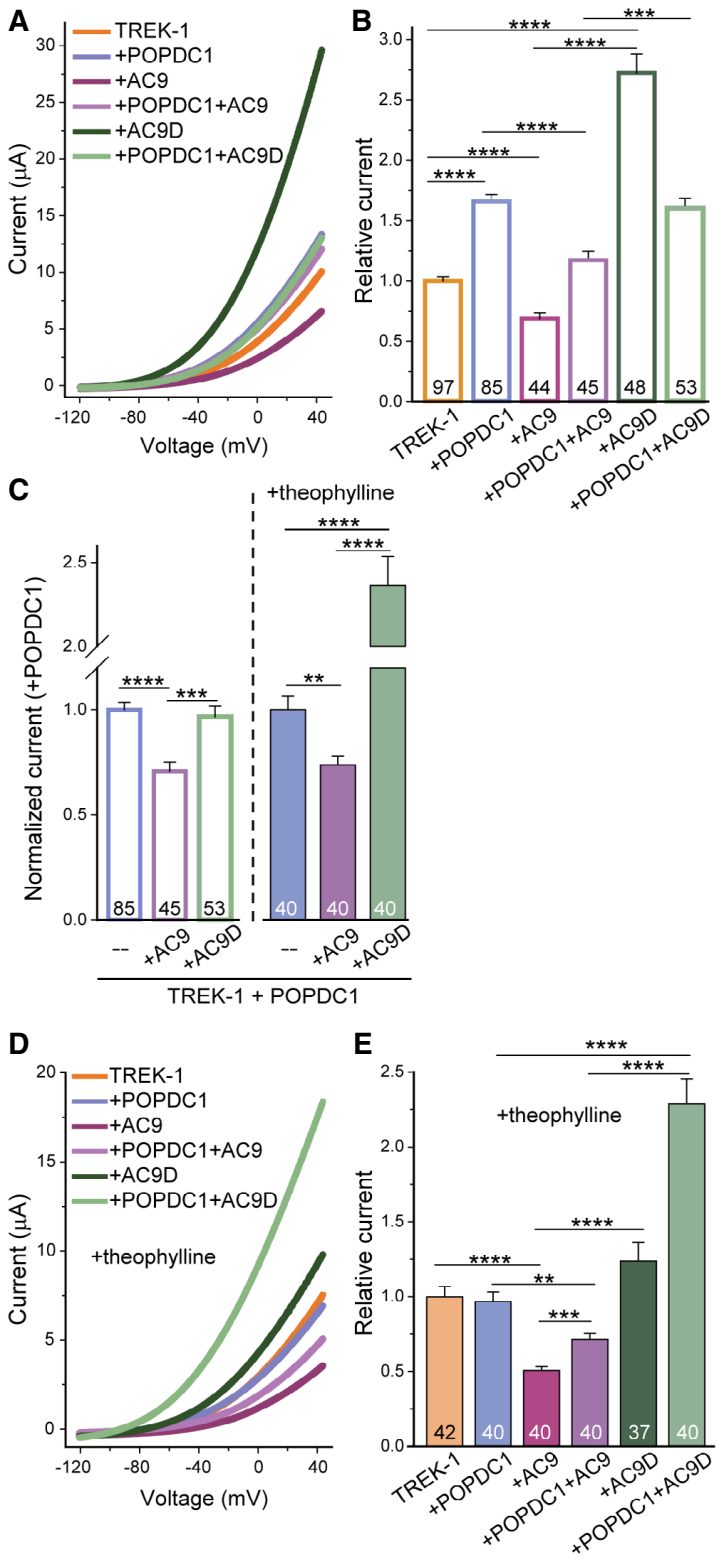
AC9 activity opposes the enhancement of TREK‐1 currents by POPDC1 ARepresentative two‐electrode voltage‐clamp measurements in *Xenopus* oocytes injected with TREK‐1 alone (orange) or TREK‐1 co‐injected with cRNAs encoding the indicated proteins. Voltage was ramped from −120 to +40 mV.BRelative TREK‐1 current amplitudes at +40 mV normalized to TREK‐1 injected alone. The number of oocytes is given within the bar graphs (*n* = 44–97). 3–5 batches of oocytes were used for each condition. Data are presented as mean ± SEM. Two‐tailed Student's *t*‐test between the indicated groups (***P = 0.00018; ****P < 1 × 10^−5^).CRelative currents normalized to TREK‐1 plus POPDC1 in the absence and presence of theophylline. Data are plotted as mean ± SEM. Two‐tailed Student's *t*‐test (**P = 0.0011; ***P = 0.0002; ****P < 1 × 10^−5^; *n* = 40–85 biological replicates).D, EOocytes were treated with 0.5 mM theophylline prior to voltage‐clamp measurements as in (A, B). Data are presented as mean ± SEM. Two‐tailed Student's *t*‐test between the indicated groups (**P = 0.0011; ***P = 4 × 10^−5^; ****P < 1 × 10^−7^). Source data are available online for this figure.

As shown previously, expression of POPDC1 promotes the membrane trafficking of TREK‐1 to the plasma membrane and thus enhances K^+^ currents when co‐expressed in *Xenopus* oocytes (Fig 8A and B; Froese *et al*, 2012). Consistent with TREK‐1 inhibition by multiple cAMP‐dependent mechanisms (Wiedmann *et al*, 2021), AC9 co‐expression reduces TREK‐1 currents (Fig 8A and B) while co‐expression with a catalytically dead form of AC9 enhances TREK‐1 currents, revealing a pool of TREK‐1 that was inactive at baseline. Co‐expression of POPDC1 with AC9 or AC9D recruits the AC to a complex with POPDC1, reducing the effects of AC9 or AC9D on baseline TREK‐1 currents. When normalized to the effects of POPDC1 alone (Fig 8C), AC9D has little effect on the POPDC1 enhancement of TREK‐1 currents, as expected given the lack of any cAMP stimulus, while AC9 reduces somewhat the POPDC1 effect. However, given the strong effects of AC9 and AC9D on background TREK‐1 currents, the interpretation of these data is complex. Therefore, we treated oocytes with the nonselective phosphodiesterase (PDE) inhibitor theophylline. As previously reported, increased cAMP by theophylline blocks any enhancement of TREK‐1 currents by POPDC1 (Fig 8D and E). In the presence of theophylline, AC9 alone inhibits TREK‐1 currents even further (0.69 ± 0.05 versus 0.51 ± 0.03; *P* = 0.001), while the enhancement by AC9D alone is suppressed by the overall elevation of cAMP by theophylline. Co‐expression of POPDC1 with AC9 once again sequesters AC9, reducing its effect on background TREK‐1 currents in the presence of theophylline. Surprisingly, co‐expression of POPDC1 with AC9D leads to synergistic enhancement of TREK‐1 currents, protecting POPDC1 from the effects of theophylline (Fig 8C and E). AC9D likely creates a local nanodomain surrounding POPDC1 lacking cAMP production and allowing POPDC1 enhancement of TREK‐1 currents. Overall, both AC9 and POPDC1 regulate TREK‐1 currents by complex cAMP‐dependent mechanisms.

## Discussion

AC9 represents the most distantly related of the mammalian AC isoforms but has clear roles in cardiac function (Marsden & Dessauer, 2019). We show that AC9 is important for resting HR control in mice. Furthermore, we identify POPDC1 as a novel adaptor that mediates interactions of AC9 with the two‐pore potassium channel TREK‐1 in an ISO‐dependent manner to mediate adrenergic regulation of membrane trafficking. POPDC1 and TREK‐1 were previously shown to be important players in HR control, while deletion of *Popdc1*, *Kcnk2*, or *Adcy9* gives rise to HR variability (Froese *et al*, 2012; Unudurthi *et al*, 2016). Our data suggest a model whereby POPDC1 recruits AC9 to a complex with TREK‐1 to enhance local cAMP within the complex and facilitate POPDC1 regulation of TREK‐1 K^+^ currents.

### POPDC1 is an adaptor for AC9 complexes

AC9 interacts with all three POPDC isoforms, as shown using a host of cellular and biochemical methods. POPDC proteins are localized in multiple membrane compartments in adult cardiomyocytes including the intercalated disk and the lateral plasma membrane, where AC9 and TREK‐1 also reside (Hund *et al*, 2014; Brand & Schindler, 2017; Li *et al*, 2019). POPDC1 can homodimerize, involving residues within the Popeye domain and likely the transmembrane domain (Kawaguchi *et al*, 2008; Russ *et al*, 2011). Given that deletion or mutation of POPDC1 often leads to loss of POPDC2 membrane localization, and deletion of either POPDC isoform closely phenocopies one another with respect to HR variability and stress‐induced bradycardia, POPDC1 likely heterodimerizes with POPDC2 (Brand *et al*, 2014; Schindler *et al*, 2016b; Brand, 2019). It is unknown if AC9 interacts only with homodimers or also with potential heterodimeric POPDC complexes.

Immunoprecipitation of scaffolding proteins followed by measurement of the associated AC activity (IP‐AC assays) have identified numerous AC:AKAP interactions in cells and tissues; however, an inactive AC was never previously pulled down with an AKAP or PKA effector protein (Piggott *et al*, 2008; Kapiloff *et al*, 2009; Efendiev *et al*, 2010; Li *et al*, 2012). We show clear interaction of POPDC1 and POPDC2 with AC9 by co‐IP but were surprised that no Gαs‐stimulated AC activity was detected in POPDC2 pull‐downs. POPDC2 may play a second, inhibitory role for AC9. Alternatively, given the possible existence of heteromeric complexes consisting of POPDC1 and POPDC2, fully active AC9 may bind to POPDC1 homodimers and possibly a POPDC1/2 heteromeric complex, while an AC9 enzyme incapable of Gαs stimulation is associated with POPDC2. The complex regulation of AC activity by POPDC proteins remains under investigation.

### TREK‐1 associates with multiple AC isoforms in both a POPDC‐dependent and independent manner in the heart

TREK‐1 channels are mechanoactivated K^+^ channels that are highly expressed throughout the human heart, including the SA and AV nodes (Schmidt *et al*, 2014; Unudurthi *et al*, 2016; Rinné *et al*, 2020). TREK‐1 is thought to regulate the resting membrane potential and therefore determines cell excitability, giving rise to a background K^+^ current in SA nodal cells (Unudurthi *et al*, 2016). TREK‐1 channels are modulated by cAMP via two distinct mechanisms (Terrenoire *et al*, 2001). TREK‐1 gating is inhibited by PKA phosphorylation of TREK‐1, which is facilitated by AKAP79 association (Noël *et al*, 2011), while TREK‐1 channel density on the plasma membrane is enhanced by POPDC1 (Froese *et al*, 2012; Rinné *et al*, 2020). However, upon POPDC1 binding of cAMP, the TREK‐1:POPDC1 complex is dissociated, leading to loss of TREK‐1 at the membrane and decreased K^+^ current (Froese *et al*, 2012).

In the heart, abundant Gαs‐stimulated AC activity is associated with TREK‐1, which is absent in cardiac tissue from *Adcy9* ^−/−^ mice, suggesting that AC9 makes up the majority of TREK‐1‐associated AC activity. Deletion of *Popdc1* abolished any TREK‐1‐associated Gαs‐stimulated AC activity, consistent with a complex that is dependent on POPDC1. Our BiFC data support this conclusion, where interactions between TREK‐1 and AC9 are not detectable in cells lacking POPDC1 expression.

The major Ca^2+^/calmodulin‐stimulated ACs, AC1, and AC8, are expressed at low levels in the SA node and possibly atria, maintaining high calcium‐stimulated cAMP levels essential for cardiac pacemaking (Mattick *et al*, 2007; Younes *et al*, 2008; Kryukova *et al*, 2012; Moen *et al*, 2019; Robinson *et al*, 2020; Capel *et al*, 2021). In the heart, TREK‐1 associates with a low amount of Ca^2+^/calmodulin‐stimulated AC activity (~ 20% of Gαs‐stimulated activity); however, this was independent of POPDC1 expression. Multiple groups have demonstrated AC8, but not AC1, binding to AKAP79 (Efendiev *et al*, 2010; Willoughby *et al*, 2010, 2012). AKAP79 is also expressed in the SA node (Vedantham *et al*, 2015) and may be mediating TREK‐1 association with Ca^2+^‐stimulated AC activity.

TREK‐1 co‐localized and bound a complex of POPDC1:AC9. Activation of endogenous beta‐adrenergic receptors by ISO decreased TREK‐1 interactions with AC9 and POPDC1 in a dose‐dependent manner. Similarly, FRET measured between POPDC1 and TREK‐1 decreases with ISO, occurring rapidly and independent of PKA activity (Froese *et al*, 2012). BiFC signals for POPDC1:AC9 were largely independent of ISO treatment, suggesting that this core complex stays intact while TREK‐1 at least partially dissociates. This model suggests differential regulation of TREK‐1 by AKAP79‐ versus POPDC1‐bound ACs. First, TREK‐1 inhibition by PKA on AKAP79 (Noël *et al*, 2011) is likely regulated by additional feedback inhibition of bound AC5, AC6, and AC8 by PKA (Baldwin & Dessauer, 2018; Musheshe *et al*, 2018; Marsden & Dessauer, 2019). Within the POPDC1:TREK‐1 complex, however, PKA regulation of AC9 has not been reported (Kawaguchi *et al*, 2008; Lolicato *et al*, 2017; Baldwin *et al*, 2019). Second, while nearly all the AKAP79‐bound cardiac ACs are stimulated by forskolin and inhibited by Gαi, AC9 is insensitive to these regulators (Kawaguchi *et al*, 2008; Lolicato *et al*, 2017; Baldwin *et al*, 2019), suggesting that the AC9:POPDC1:TREK‐1 complex is not regulated by Gi‐coupled receptors. Third, if the association of Ca^2+^‐stimulated AC activity with TREK‐1 in heart is due to AC8:AKAP79 scaffolding, then inhibition of TREK‐1 within AKAP, but not POPDC1, complexes would be Ca^2+^‐sensitive. Finally, the nanometer size of cAMP domains and the failure of these domains to cross‐talk with other GPCRs at low agonist concentrations suggests that AKAP and POPDC1 molecular complexes dictate the specificity of cellular responses regulating TREK‐1 (Anton *et al*, 2022).

The complexity of multiple cAMP‐dependent mechanisms regulating TREK‐1 currents is even evident in model systems such as *Xenopus* oocytes, which display PKA‐dependent inhibition of TREK‐1 and express endogenous POPDC1 (Lopes *et al*, 2007; Claußen *et al*, 2015; Wiedmann *et al*, 2021). Expression of catalytically inactive AC9D with TREK‐1 greatly enhances TREK‐1 currents, likely by competing with endogenous ACs for association with AKAPs and POPDC1. AC9 and AC9D have opposing effects on TREK‐1 currents at baseline and on POPDC1 enhancement of TREK‐1. This is particularly evident in the presence of theophylline, where recruitment of AC9D to POPDC1 protects the complex from the effects of theophylline, allowing for greatly enhanced TREK‐1 currents by POPDC1. Local effects of scaffolded AC9 have been observed previously for KCNQ1, as isoproterenol‐stimulated PKA phosphorylation of KCNQ1 is abolished in the presence of AC9D (Li *et al*, 2012, 2017, 2019). Similarly binding of AC9 to the POPDC1:TREK‐1 complex appears to control the level of cAMP within the complex to regulate TREK‐1 currents.

### AC9, POPDC1, and TREK‐1 participate in heart rate control

We previously reported a bradycardia in *Adcy9* ^−/−^ mice under anesthesia (Li *et al*, 2017). Using telemetry, a bradycardia was revealed at rest during daytime when the animals are naturally less active. This was not due to AC9 regulation of *I*_Ks_ currents, which are absent in adult WT mice (Li *et al*, 2017) but may be due to increased TREK‐1 currents, which would stabilize the membrane potential in the SA node. *Adcy9* ^−/−^ mice also displayed an increase in HR variability during the recovery period from high stress, as mimicked by beta‐adrenergic stimulation upon ISO injection. However, during exercise or immediately following ISO injection, the increased HR in *Adcy9* ^−/−^ mice was similar to wild‐type. This is consistent with the lack of AC9 regulation of global PKA phosphorylation of targets such as CREB, troponin I, or phospholamban in response to ISO injection (Li *et al*, 2017). Similarly, loss of AC9 decreases the PKA‐dependent phosphorylation of the heat shock protein 20 at baseline, but not upon ISO injection. Thus, AC9 represents only a small percentage of AC activity in heart and functions largely at baseline or within local scaffolded complexes (Li *et al*, 2017, 2019).

TREK‐1 is also important for resting HR, as a cardiac‐specific deletion of *Kcnk2* in mice exhibits bradycardia with frequent sinus pauses (Unudurthi *et al*, 2016). TREK‐1 downregulation has been further implicated in atrial fibrillation, while a heterozygous point mutation of TREK‐1 is linked to idiopathic ventricular arrhythmia (Wiedmann *et al*, 2021). Mutant mice with a deletion of either *Popdc1* or *Popdc2* exhibit severe stress‐induced bradycardia with high HR variability and long sinus pauses (Froese *et al*, 2012). In patients, missense or nonsense mutations of *POPDC1* that affect cAMP binding or impair membrane trafficking lead to a host of cardiac and skeletal muscle‐associated phenotypes (Brand, 2019). For example, patients carrying the mutations *POPDC1 p.S201F* or *POPDC2 p.W188X* show decreased cAMP binding to POPDC, impaired TREK‐1 regulation, and develop an AV block in addition to other phenotypes (Schindler *et al*, 2016b; Rinné *et al*, 2020). The functional conservation of cardiac arrhythmia phenotypes of *POPDC1* mutations from zebrafish, mice, and humans suggests that the identified POPDC1 complexes are likely relevant in regulating cardiac function in the human heart as well. However, it is unlikely that the impaired regulation of TREK‐1 alone is sufficient to explain the strong phenotype of the *Popdc1* and ‐*2* null mutants, particularly when compared to phenotypes of *Kcnk2* or *Adcy9* deletions in mice. Recently, an interaction of POPDC1 and PDE4 isoforms has been described (Tibbo *et al*, 2022). A cell‐permeable peptide that disrupts the POPDC1:PDE4 complex caused a reduction in the cycle length of spontaneous Ca^2+^ transients in mouse SA nodes. This effect of the peptide was only seen at baseline and was blunted after ISO stimulation. It will be important to determine whether PDE4 is part of the same scaffold as AC9 and TREK‐1. POPDC proteins interact with several additional cardiac proteins, including caveolin‐3 (Alcalay *et al*, 2013) and the sodium‐calcium exchanger (NCX1; Lubelwana Hafver *et al*, 2017), that are important for heart rate control (Torrente *et al*, 2015; Vaidyanathan *et al*, 2018). Whether caveolin‐3 or NCX1 is also part of the AC9:POPDC1 complex needs to be tested. In conclusion, POPDC proteins represent a unique cAMP effector and AC9 adaptor protein that bridge interactions with TREK‐1 to regulate heart rate.

## Materials and Methods

### Gene‐targeted mice

*Adcy9* ^−/−^ mice were obtained from the Mutant Mouse Regional Resource Center. The mouse strain B6;129S5‐Adcy9Gt(neo)159Lex/Mmucd, identification number 011682‐UCD contains an insertion of the gene trap vector between exons 1 and 2 of *Adcy9*; generation, backcrossing, and genotyping of mice are further described in (Li *et al*, 2017). *Adcy9* ^−/−^ mice show a preweaning sub‐viable homozygous phenotype with incomplete penetrance. To obtain the necessary animal numbers, crosses were set up with *Adcy9* ^−/−^ x *Adcy9* ^+/−^ versus WT x *Adcy9* ^+/−^. Age‐matched, wild‐type C57BL/6J controls were used for all behavioral experiments. Animals were housed at 22°C with a 12 h light/dark cycle (7 am–7 pm) with free access to water and irradiated chow diet. For exercise experiments, animals were not pretrained to run. All *Adcy9* ^−/−^ animal protocols were approved by the Institutional Animal Care and Use Committee (IACUC; 18‐0095) at the University of Texas Health Science Center at Houston in accordance with the Animal Welfare Act and NIH guidelines. *Popdc1* ^−/−^ mice were generated by the replacement of the first coding exon with a β‐galactosidase‐encoding gene cassette as described (Andree *et al*, 2002). The *Popdc1* ^−/−^ mice were backcrossed and maintained on a C57BL/6J background. Wild‐type controls for biochemical assays of hearts from *Adcy9* ^−/−^ or *Popdc1* ^−/−^ mice used age‐matched and/or littermates from their respective breeding facilities. Work with *Popdc1* ^−/−^ animals were approved by the Animal Welfare and Ethical Review Board of Imperial College London.

### Behavioral assays

All behavioral tests were performed by blinded investigators and randomized in groups by an investigator not involved in testing. Mice were acclimated to the behavioral testing room for 30–60 min in their home cages with the investigator present before beginning testing. Elevated plus maze and open field activity box were performed 1 week prior and again after telemetry implantation surgery.

### Elevated plus maze

The 40 cm high EPM was used to measure differences in anxiety. It consisted of four, 12 cm wide arms: two enclosed by 40 cm high walls and two open (modeled upon the Stoelting Co′s EPM model, Wood Dale, IL). Individual mice were placed in the EPM center facing an open arm and explored freely for 5 min. The test was video recorded and later analyzed for time spent in the open versus closed arms by an investigator blinded to genotypes, as previously described (Pellow *et al*, 1985; Acharjee *et al*, 2013; Nyuyki *et al*, 2018).

### Open field activity box

The open field box (ENV‐515, Med Associates, Inc., St. Albans, VT) consists of an activity chamber (43.2 cm wide by 43.2 cm deep by 30.5 cm high) with 16 infrared transmitters and receivers evenly positioned around the chamber's periphery. Mice placed in the activity chamber moved freely for 25 min. Data were collected by the Open Field Activity (OFA) software that registers time spent and movements within the chamber as a whole versus the inner zone 1 by recording photobeam interruptions.

### ECG and telemetry

ECGs were recorded using an ETA‐F10 ECG transmitter (DSI, St. Paul, MN) implanted into 10 wild‐type and 10 *Adcy9* ^−/−^ mice (6 males and 4 females per group; one male WT mouse removed the transmitter lead and was dropped from all telemetry analysis). Mice were allowed to recover for at least 1‐week postsurgery. ECGs were recorded in mice 5–8 months of age (average weight minus probe = 24.4 ± 2.7 g). Telemetric ECG was recorded during normal activity and during the following stress tests: free running wheel, forced swim test, and ISO injections (methods described below). ECG data were analyzed in Ponemah v6.52 (DSI, St. Paul, MN). HR and RR intervals were averaged from Ponemah exported data. Telemetric ECGs were manually analyzed with respect to arrhythmias by examiners blinded to the genotypes and corrections made for any beats not correctly identified by the automated software analysis. HR variability was calculated in Ponemah using the Variability Analysis Time Domain function and averages were determined from the exported data from Ponemah. pNNx value was set at 6 ms, as is standard for mice.

### Running wheel

Mice were placed in cages for 24 h containing voluntary running wheels fitted with electronic monitors for activity tracking by Activity Wheel Monitor software (Lafayette Instruments). ECGs were recorded throughout the 24 h period. Distance traveled on wheels was cross‐referenced with the HR data to calculate HR during active versus inactive and recovery periods. Active times were defined as sustained activity on the wheel for 5 min and selected by examiners blinded to the genotypes. Inactive times were selected only if the wheel did not move for a full 10 min. Active and inactive times were selected for analysis by blinded investigators.

### Forced swim test

Forced swim tests were conducted in 5 liter beakers filled with autoclaved water (26–28°C) to 16 cm according to published protocols (Petit‐Demouliere *et al*, 2005). Mice were placed in water containers for 6 min; recorded swimming behavior was quantified during the last 4 min by blinded investigators.

### Isoproterenol (ISO) Injections

ECGs were recorded for at least 1 h prior to injections. ISO (1 μg drug per g weight of mouse; subtracting probe weight) was administered by intraperitoneal injection. Mice were returned to cages for at least 1 h and 30 min post‐ISO injection for ECG recordings.

### Plasmids

Flag‐ and YFP‐tagged AC9 pCDNA3 plasmids are as described (Li *et al*, 2017; Lazar *et al*, 2020). POPDC1‐Myc, POPDC2‐Myc, POPDC1Δ172‐Myc, and POPDC1Δ236‐Myc were described in (Froese *et al*, 2012; Schindler *et al*, 2016b). Human ADCY9 cDNA and a catalytically inactive ADCY9 D399A (AC9D; Li *et al*, 2017) were subcloned into the BamHI‐XhoI sites of the psGEM vector for expression in *Xenopus* oocytes. Bimolecular Fluorescence complementation constructs consist of split Venus fluorescent protein. The N‐terminal (VN) or C‐terminal (VC) half of Venus was fused to the C‐terminus of mouse POPDC1, POPDC2, POPDC3, and POPDC1Δ236, and the N‐terminus of human TREK‐1c. In each case, previously described tagged constructs of each protein were used as the starting point for cloning, whereby the existing tag was swapped for a BiFC tag by PCR (Froese *et al*, 2012; Schindler *et al*, 2016b). AC9‐VN, AC9‐VC, and AC9D‐VC lack N‐terminal tags and contain a 7 aa linker (AAAGGGS) between AC9 and VN or VC, as described (Baldwin *et al*, 2019). To create the myristoylated cytoplasmic domain of POPDC1, the myristoylation and palmitoylation sequence from Lyn was fused to aa 117 of POPDC1 (starting immediately after the three membrane‐spanning regions); an MYC or VN tag is contained at the C‐terminus. All constructs were verified by DNA sequencing.

### Cell culture and transfections

HEK293 (human, CRL‐1573) and COS‐7 (African green monkey, CRL‐1651) cells were authenticated by ATCC, cultured in Dulbecco's Modified Eagle Medium with 10% fetal bovine serum, and transfected with the indicated plasmids using Lipofectamine 2000 (Piggott *et al*, 2008; Efendiev *et al*, 2010). ISO was stored and diluted in AT buffer (100 mM ascorbate and 10 mM thiourea, pH 7.4) for cellular studies. Neonatal rat cardiomyocytes (CMs) were isolated as previously described (Li *et al*, 2019). CMs were enriched via percoll gradient centrifugation and cultured in complete media (40% DMEM, 40% HAMS/F10, 20% FBS, 1% P/S).

### Proximity ligation assay

*In situ* PLA was performed using a Duolink kit (Sigma‐Aldrich, cat. DUO92101) following the manufacturer's protocol. HEK293 cells were cultured on clear bottom 96‐well plates (Greiner Bio‐One), transfected with the required plasmids, and fixed with 4% PFA. After washing the plate 3 times with PBS, the cells were blocked (1% BSA + 0.075% Triton X100) for 1 h at room temperature and then incubated with primary antibodies overnight. Antibodies included: rabbit anti‐GFP (SC‐8334, SantaCruz, 1:1,000) and mouse anti‐MYC (purified by National Cell Culture from the ATCC hybridoma CRL‐1729 for MYC 1‐9E10.2, 1:1,000) or rabbit anti‐Gβγ (SC‐378, SantaCruz, 1:1,000) with mouse anti‐GFP (Clonetech (JL‐8), 1:1,000). After removal of primary antibodies, the samples were incubated with anti‐mouse PLUS and anti‐rabbit MINUS PLA probes for 1 h at 37°C. Subsequent steps of ligation and amplification were according to the manufacturer's protocol. After the last wash, cells were stained with DAPI (1 μg/ml) and imaged using an epifluorescence high content imaging microscope with a 20X objective, 25 fields of view per condition (> 2,000 cells per condition per experiment; CellInsight CX5 High Content Screening platform, ThermoFisher). Data analysis of mean fluorescence intensity per cell was performed using FACS analysis software (FlowJo, USA). Each experimental condition was repeated at a minimum in 3 separate experiments. To prevent false positives, cells with saturating YFP fluorescence were not considered in the analysis.

For PLA in rat neonatal CMs, cells were plated in 96‐well plates (1.8 × 10^4^ CMs per well) and infected 48 h postisolation with adenoviruses expressing Flag‐tagged GFP versus Flag‐tagged AC9 (multiplicity of infection = 10). CMs were used 65 h later for PLA as described above. Primary antibodies included: mouse anti‐FLAG (Cell Signaling Technology (9A3) 8146, 1:1,000) and rabbit anti‐POPDC1 (Sigma HPA014788, 1:500), rabbit anti‐POPDC2 (Sigma HPA024255, 1:500), or rabbit anti‐Gβγ (SC‐378, SantaCruz, 1:1,000). POPDC1 and POPDC2 antibodies were tested with sections of WT and *Popdc1* and *Popdc2* null mutants and found to be nonreactive in case of null mutant tissue (Alcalay *et al*, 2013; Schindler et al unpublished). Cells were imaged and analyzed using > 1,500 cardiomyocytes per condition per experiment. Signal was normalized to control GFP‐Gβγ signal to account for background variability between experiments.

### Bimolecular fluorescence complementation (BiFC)

Cells were plated in uncoated 12‐well plates 1 day prior to transfection with Lipofectamine 2000. 48 h post‐transfection, cells were washed once with PBS and then incubated with Tyrode's buffer at 37°C for 10 min. Where indicated, cells were treated with or without ISO at 37°C for an additional 10 min. Cells were harvested and transferred to a 96‐well plate; an aliquot of each condition was used for total protein determination by Bradford analysis. BiFC (Venus) signals were obtained using a Tecan fluorescence microplate reader at excitation wavelength 506 nm and emission wavelengths 538–542 nm as described (Baldwin *et al*, 2019). Background fluorescence was averaged from multiple wells with cells expressing empty vector control (pCDNA3) and proteins tagged with VN or VC in the absence of the other Venus half. BiFC was calculated as YFP signals minus average background signal and normalized to total protein for each sample in HEK293 cells. For COS‐7 cells, 48 h post‐transfection, cells were washed and incubated with DMEM containing DAPI (10 μg/ml) for 1 h at 37°C. Cells were washed, transferred in PBS to 96‐well plates, and YFP and DAPI (358 nm excitation and 461 nm emission) signals were measured at room temperature. YFP signals were normalized to DAPI staining after subtraction of average background YFP fluorescence from multiple control samples. Each condition per experiment represents an average of 3–4 wells with > 10,000 cells per well.

### Live‐cell imaging

For BiFC imaging, HEK293 cells were plated on Matek dishes coated with poly‐lysine. Prior to imaging, media was replaced with Tyrode's buffer (10 mM HEPES, pH 7.4, 145 mM NaCl, 4 mM KCl, 1 mM MgCl2, 1 mM CaCl_2_, 10 mM D‐Glucose). BiFC images were acquired using a TE 2000 microscope (Nikon, Tokyo, Japan) with a DG4 xenon light source and CoolSNAP cameras (Roper Scientific, Trenton, NJ). Venus fluorescent protein images were acquired 36–48 h after transfection (excitation 500/20 nm, emission 535/30 nm).

### FLIM‐FRET

FLIM experiments were performed with a lifetime fluorescence imaging attachment (Lambert Instruments, Leutingewolde, the Netherlands) on an inverted wide‐field microscope (Nikon). Cells plated on poly‐lysine coated Matek dishes and transfected as indicated were excited by using a sinusoidally modulated 3‐W 448‐nm light‐emitting diode at 40 MHz under epi‐illumination. Fluorescein at the concentration of 1 μM was used as a lifetime reference standard (4 ns). Cells were imaged with a 40x Plan‐Apo oil‐immersion objective (numerical aperture 1.45) using a CFP filter set; exposure times were less than 150 ms to avoid photobleaching. The phase and modulation lifetimes were determined from a set of 12‐phase settings using the manufacturer's software (LIFA). FRET efficiency was calculated according to the formula F_eff_ = 1 − τ2/τ1. FLIM data were averaged on a per‐cell basis. Three experiments (> 40 cells in total) were performed for each condition. Donor alone and donor‐acceptor measurements were taken immediately after one another. In order to prevent differences in donor lifetimes due to altered lipid environments, Cerulean‐TREK‐1 lifetime was measured in the presence of POPDC1 (tagged with Myc or Venus) to enhance PM localization.

### Immunoprecipitations and Western blotting

Antibodies and reagents used for immunoprecipitation and western blotting include mouse anti‐FLAG M2 agarose affinity gel (Sigma‐Aldrich), mouse anti‐DYKDDDDK (Flag) tag (Cell Signaling Technologies, Danvers, MA), mouse anti‐MYC (purified by National Cell Culture from the ATCC hybridoma CRL‐1729 for MYC 1‐9E10.2), mouse anti‐A.v. monoclonal antibody for green fluorescent protein (JL‐8; Takara Bio, Kusatsu, Japan; recognizes VC), rabbit anti‐GFP (D5.1 Cell Signaling Technology 2956S; recognizes VN), mouse anti‐β‐actin (C4, Santa Cruz Biotechnology), anti‐TREK‐1 (Neuromics, Edina, MN), anti‐POPDC1 (early studies used Santa Cruz, goat #sc‐49889 but now discontinued; Sigma‐Aldrich, rabbit #HP018176), and normal mouse or rabbit IgG (Santa Cruz Biotechnology). The rabbit anti‐AC9 antibody was generated and characterized as described (Baldwin *et al*, 2019). Note, POPDC protein runs as multiple bands (48–65 kDa) on western blots with altered sizes/patterns in different tissues due to changes in glycosylation patterns (Schindler *et al*, 2016a). AC9 generally runs as a single band in Sf9 cells but as multiple bands in mammalian cell lines due to variations in glycosylation and dimerization.

### Adenylyl cyclase activity and immunoprecipitation‐AC assays

Preparation of heart extracts and measurement of AC activity was performed as previously described (Piggott *et al*, 2008; Efendiev *et al*, 2010). Immunoprecipitation of POPDC complexes followed by western blotting or measurement of associated AC activity (IP‐AC assay) was performed as described (Li & Dessauer, 2015). AC activity was stimulated with the indicated reagents or proteins (purified and activated GTPγS‐Gαs or calcium and purified calmodulin) and cAMP was detected by enzyme immunoassay (Assay Designs) or using [γ^32^P]ATP.

### Voltage‐clamp measurement of TREK‐1 current

*Xenopus laevis* toads were held and ovarian lobes were surgically removed in order to enzymatically isolate oocytes, as previously described (Putzke *et al*, 2007). Capped cRNA transcripts were synthesized *in vitro* and photometrically quantified. Oocytes were injected with 50 nl of cRNAs encoding human hTREK‐1b (Acc. Nr: AF004711; 0.25 ng), alone or together with mouse POPDC1 (1.25 ng) and/or human AC9 cRNA (1.25 ng) or human AC9D cRNA (1.25 ng). The injected oocytes were stored at 19°C in ND96 recording solution (96 mM NaCl, 2 mM KCl, 1 mM MgCl_2_, 1.8 mM CaCl_2_, and 5 mM HEPES (pH 7.4–7.5) supplemented with 50 mg/l gentamicin and 275 mg/l sodium pyruvate) and assayed 48 h postinjection. Theophylline (0.5 mM) was supplemented to the storage solution, directly following the cRNA injection, where indicated. Standard two‐electrode voltage‐clamp recordings were performed at room temperature (21–22°C) as described (Putzke *et al*, 2007; Schindler *et al*, 2016b). Since current amplitudes vary by nature from one batch of oocytes to the next, currents were normalized to TREK‐1 WT current amplitudes of the respective batch and recording day.

### Statistical analysis

Sample sizes for WT and *Adcy9* ^−/−^ mice for telemetry were based upon previously measured bradycardia using doppler imaging in mice 5–7 months of age (*P* = 0.0002, *n* = 10; Li *et al*, 2017). Repeated‐measures ANOVA was used for testing the significance in the HR and HR variability data with multiple data points over time. Data presented as boxplots show the median as the central band, the box size as the lower and upper quartiles, while the 10th and 90th percentiles are represented by the whiskers (error bars). Data are expressed as mean ± standard error of the mean (SEM), except for behavioral data in Table I, which are expressed as mean ± the standard deviation (SD). Normality was determined by the Shapiro–Wilk test. Differences between samples were determined using one‐way or two‐way analysis of variance (ANOVA) followed by the indicated tests for comparison between multiple groups. For comparisons between two groups with equal variance, a two‐sided Student's *t*‐test was used. For samples of nonequal variance, the nonparametric Kruskal–Wallis ANOVA on ranks or Mann–Whitney rank‐sum test was performed. Significant *P*‐values are indicated as follows: (*) denotes a *P*‐value < 0.05, (**) < 0.01 and (***) < 0.001. All analyses were performed using SigmaPlot statistical analysis software with the exception of electrophysiological recordings, which used the Student's two‐sided *t*‐test in Excel.

## Author contributions

**Tanya A Baldwin:** Conceptualization; formal analysis; investigation; methodology; writing – review and editing. **Yong Li:** Formal analysis; supervision; investigation; methodology; writing – review and editing. **Autumn Marsden:** Formal analysis; investigation; methodology; writing – review and editing. **Susanne Rinne:** Formal analysis; investigation; writing – review and editing. **Anibal Garza Carbajal:** Formal analysis; investigation; methodology. **Roland FR Schindler:** Conceptualization; resources; methodology. **Musi Zhang:** Investigation. **Mia A Garcia:** Investigation. **Venugopal R Venna:** Supervision; methodology; writing – review and editing. **Niels Decher:** Conceptualization; formal analysis; supervision; funding acquisition; project administration; writing – review and editing. **Thomas Brand:** Conceptualization; resources; supervision; funding acquisition; project administration; writing – review and editing. **Carmen W Dessauer:** Conceptualization; formal analysis; supervision; funding acquisition; investigation; writing – original draft; project administration.

## Disclosure and competing interests statement

The authors declare that they have no conflict of interest.

## Supporting information




Appendix

Expanded View Figures PDF

Source Data for Figure 1

Source Data for Figure 2

Source Data for Figure 3

Source Data for Figure 4

Source Data for Figure 5

Source Data for Figure 6

Source Data for Figure 7

Source Data for Figure 8

Source Data for Table 1

PDF+

## Data Availability

This study includes no data deposited in external repositories.
