# Supplementary material for: POPDC1 scaffolds a complex of adenylyl cyclase 9 and the potassium channel TREK‐1 in heart
Source: EMBO Rep. 2022 Oct 18;23(12):e55208. doi: 10.15252/embr.202255208 (PMC9724675; doi:10.15252/embr.202255208)
Supplement: Supplementary file 1 — Appendix [file EMBR-23-e55208-s009.pdf]

Popeye Domain-Containing Protein 1 Scaffolds a Complex of Adenylyl Cyclase 9 and the Two-Pore-Domain Potassium Channel TREK-1 in Heart

Tanya A. Baldwin[1†], Yong Li[1†], Autumn Marsden[1], Susanne Rinné[4], Anibal Garza Carbajal[1], Roland F.R. Schindler[3], Musi Zhang[1], Mia A. Garcia[1], Venugopal Reddy Venna[2], Niels Decher[4], Thomas Brand[3], and Carmen W. Dessauer[1*]

[1]Dept. Integrative Biology and Pharmacology, McGovern Medical School, University of Texas Health Science Center, Houston, TX 77030

[2]Dept. Neurology, McGovern Medical School, University of Texas Health Science Center, Houston, TX 77030

[3]National Heart and Lung Institute, Imperial College London, London, United Kingdom

[4]Institute for Physiology and Pathophysiology, Vegetative Physiology and Marburg Center for Mind, Brain and Behavior MCMBB, Philipps-University of Marburg, Marburg, Germany

[†]These authors contributed equally to this work.

*Corresponding author. Tel: +1 713-500-6308. Carmen.W.Dessauer@uth.tmc.edu

Appendix Table of Context

Appendix Table S1:

Appendix Table 1. Expression of POPDC1 and POPDC2 RNA in HEK293 cells is 10-fold greater than in COS-7 cells. ……..Page 2

Appendix Figures:

Appendix Figure S1. Immunoprecipitation of TREK-1 pulls down POPDC1 and AC9. …..Page 2

Appendix Figure S2. Validation of anti-TREK-1 antibody for immunoprecipitation. ……..Page 3

Appendix Figure S3. Western blot examples of HEK293 lysates expressing BiFC constructs with and without ISO stimulation. ……..Page 3

Appendix Table S1. Expression of POPDC1 and POPDC2 RNA in HEK293 cells is 10-fold greater than in COS-7 cells.

RT-PCR of POPDC isoforms for HEK393 versus COS-7 cells and normalized using either GAPDH or 18S rRNA.

	Fold change* (GAPDH)	Fold change* (18S rRNA)
POPDC1	14.4	15.9
POPDC2	9.4	13.1

*(2^ΔCT, HEK293 vs COS-7)

Primer sequences (conserved in human and African green monkey):

POPDC1, Forward: 5'- ATGAAGGTCTCCTATCGAGGAC-3'

POPDC1, Reverse: 5'-CTGCAGTTATCATCTGCAATAATG-3'

POPDC2, Forward: 5'- GCAGTTTCTGCACTACATCTTTC-3'

POPDC2, Reverse: 5'-GATGTATCGCTCTTTGGTCAGAA-3'

Appendix Figures

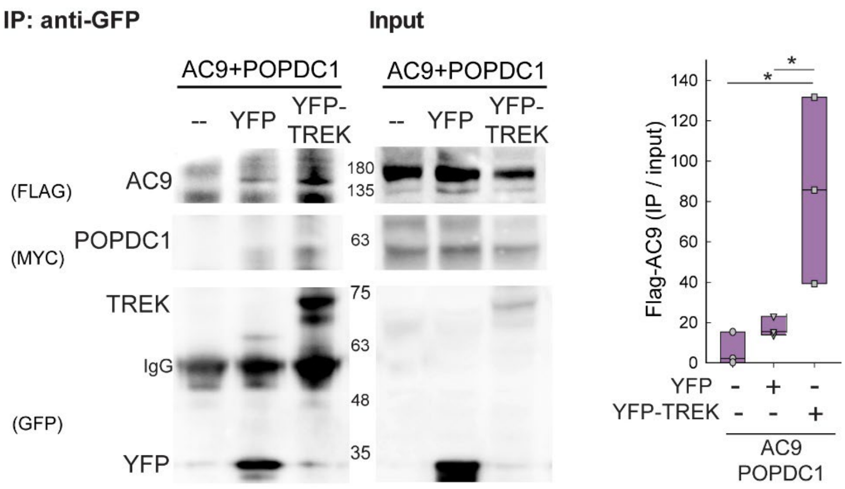

Appendix Figure S1. Immunoprecipitation of TREK-1 pulls down POPDC1 and AC9. Performed as indicated in Fig 4D, comparing YFP expression alone versus YFP-tagged TREK-1. Quantification (IP/input) of Flag-AC9 pulldown for the different samples is shown on the right. Kruskal-Wallis One Way ANOVA analysis of Variance on Ranks was performed (n=3 experiments; P=0.01 overall) with multiple comparisons by Student-Newman-Keuls Method (*P<0.05). Boxplots show the median as the central band, the box size as the lower and upper quartiles, while the 10th and 90th percentiles are represented by the whiskers.

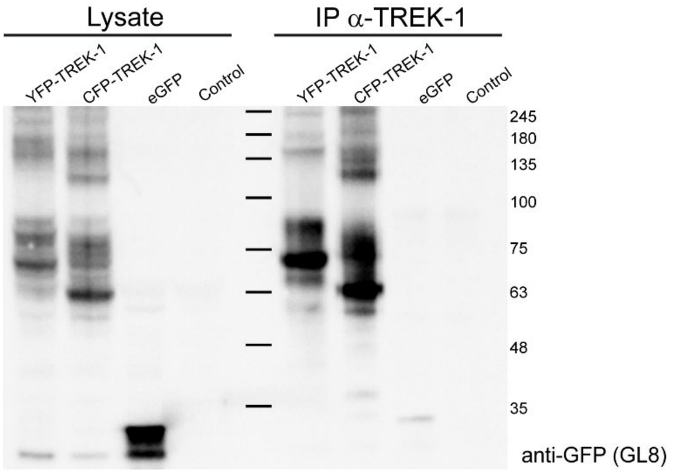

Appendix Fig 2. Validation of anti-TREK-1 antibody for immunoprecipitation. GFP, YFP-tagged TREK-1, and CFP-tagged TREK-1 were expressed in HEK293 cells and immunoprecipitated with TREK-1 antibody. Cell lysates and immunoprecipitates were then subjected to western blotting with anti-GFP. Anti-TREK-1 pulled down tagged TREK-1 proteins but not GFP controls.

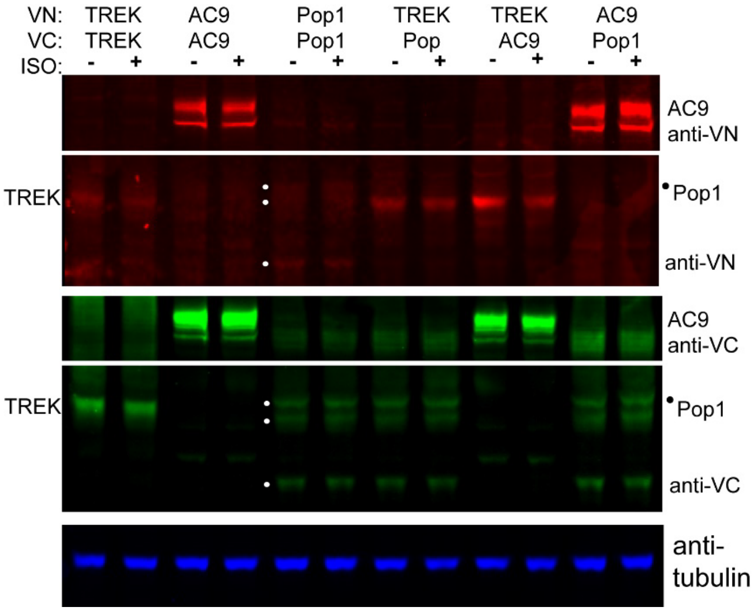

Appendix Fig 3. Western blot examples of HEK293 lysates expressing BiFC constructs with and without ISO stimulation. As shown in Fig 5B, indicated constructs were expressed in HEK293 cells and treated with isoproterenol. Cell lysates were subjected to western blotting using anti-VN (Gfp (D5.1) XP® Rabbit mAb) or anti-VC (JL-8 mouse monoclonal) antibodies against GFP.
