# Supplementary material for: POPDC1 scaffolds a complex of adenylyl cyclase 9 and the potassium channel TREK‐1 in heart
Source: EMBO Rep. 2022 Oct 18;23(12):e55208. doi: 10.15252/embr.202255208 (PMC9724675; doi:10.15252/embr.202255208)
Supplement: Supplementary file 2 — Expanded View Figures PDF [file EMBR-23-e55208-s006.pdf]

Expanded View Figures

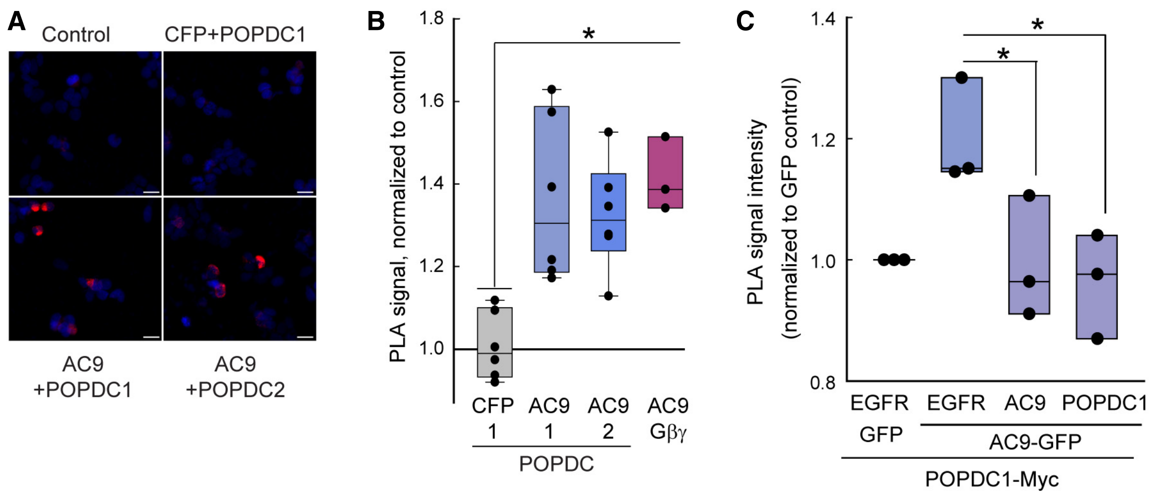

Figure EV1. PLA signal between GFP-tagged AC9 and POPDC1-Myc.

A, B Images (A) of PLA signal (red) and DAPI (blue) performed in HEK293 cells using antibodies against GFP and MYC tags. Scale bars are 20 μm. Mean cellular fluorescence intensity of PLA signal (B) was quantified by high content microscopy. The Kruskal–Wallis one-way ANOVA analysis was performed ($n = 7$ experiments, $P = 0.003$ between groups) with multiple comparisons by the Bonferroni t-test (*$P < 0.05$). Boxplots show the median as the central band, the box size as the lower and upper quartiles, while the 10th and 90th percentiles are represented by the whiskers.

C Competition of PLA signal between GFP-tagged AC9 and POPDC1-Myc with overexpression of nontagged EGFR (control), Flag-AC9, or POPDC1-Flag in HEK293 cells. Mean cellular fluorescence intensity was quantified by high content microscopy and normalized to GFP control. The Kruskal–Wallis one-way ANOVA analysis was performed ($n = 3$ experiments, >1,000 cells per experiment) with multiple comparisons by the Student–Newman–Keuls method (*$P < 0.05$). Boxplots show the median as the central band, the box size as the lower and upper quartiles, while the whiskers are the range.

 ©2022 The Authors
