# Supplementary material for: POPDC1 scaffolds a complex of adenylyl cyclase 9 and the potassium channel TREK‐1 in heart
Source: EMBO Rep. 2022 Oct 18;23(12):e55208. doi: 10.15252/embr.202255208 (PMC9724675; doi:10.15252/embr.202255208)
Supplement: Supplementary file 13 [file EMBR-23-e55208-s004.pdf]

POPDC1 Scaffolds a Complex of Adenylyl Cyclase 9 and the Potassium Channel TREK-1 in Heart

Tanya Baldwin, Yong Li, Autumn Marsden, Susanne Rinne, Anibal Garza Carbajal, Roland Schindler, Musi Zhang, Mia Garcia, Venugopal Venna, Niels Decher, Thomas Brand, and Carmen W. Dessauer
DOI: 10.15252/embr.202255208

Corresponding author(s): Carmen W. Dessauer (Carmen.W.Dessauer@uth.tmc.edu)

Review Timeline:

Transfer from Review Commons:	8th Apr 22
Editorial Decision:	13th Apr 22
Revision Received:	27th Jul 22
Editorial Decision:	20th Sep 22
Revision Received:	23rd Sep 22
Accepted:	29th Sep 22

Editor: Martina Rembold/ Deniz Senyilmaz-Tiebe

Transaction Report: This manuscript was transferred to EMBO Reports following peer review at Review Commons.

(Note: With the exception of the correction of typographical or spelling errors that could be a source of ambiguity, letters and reports are not edited. Depending on transfer agreements, referee reports obtained elsewhere may or may not be included in this compilation. Referee reports are anonymous unless the Referee chooses to sign their reports.)

Review #1

1. Evidence, reproducibility and clarity:

Evidence, reproducibility and clarity (Required)

Summary:

The authors have a novel adenylyl cyclase (AC) scaffold, the Popeye domain-containing (POPDC) protein. They demonstrate that unlike other currently known AC scaffolding proteins such as AKAPs, POPDC1 binds cAMP with high affinity. As the POPDC family of proteins have been implicated in cardiac pacemaking and conduction, in part because of cAMP-dependent regulation of TREK-1 potassium channels. The authors next show that TREK-1 binds the AC9:POPDC1 complex and co-purifies with AC9-associated activity in heart in way that depends on the presence of POPDC1. They note that the interaction of AC9 and POPDC1 was cAMP independent. However, TREK-1 association with AC9/POPDC1 was reduced in an isoproterenol-dependent manner, requiring both an intact cAMP binding Popeye domain and AC activity in the complex. Finally loss of AC9 in a transgenic mouse model led to bradycardia at rest and isoproterenol-induced heart rate variability, a phenotype previously seen following loss of Popdc1, and similar to loss of TREK-1.

Major Comments:

1) The authors start, convincingly with in vivo experiments. I feel that for the non-specialist reader, the parameters associated with their analysis, RR interval and methods such as RMSSD and pNN6 could be better explained.

2) PLA and BiFC experiments in Figure 2. It is good that the authors used multiple techniques to demonstrate the interaction and in general I acknowledge that a paucity of good antibodies is a legitimate technical issue in studies such as this. However, some additional controls might be suggested that involve competition of the PLA or BiFC signals by untagged competitor proteins. Also, are the proteins tagged still functional? The results shown with different POPDCs are not entirely consistent between techniques- is this related to loss of functionality in some sense?

3) In the IP-AC assays, POPDC1 and POPDC2 have differential responses to Gs stimulation yet both still are associated with the complex. What is the significance of this?

4) With regard to demonstrating the interaction in homologous systems, the authors rightly bring up the absence of good antibodies. Could they express single tagged versions of these proteins in cardiomyocytes and pull down the tag combined with M/S analysis to show that the other proteins are associated in cells other than HEK 293 cells or other heterologous systems?

5) In Figure 6, is there some way to assess the effect isoproterenol or FSK stimulation on the amount of TREK pulled down?

6) Is there some way to assess the effect of this complex on TREK channel activity in a heterologous system?

7) Might we expect that PKA is involved here as well or does cAMP binding act as the primary determinant of complex dissocation? Is there a concomitant role for Gi-coupled receptors in upregulating the activity of the complex? Are the effects they measured phenocopied by other Gs-coupled receptors or direct activation of AC with forskolin?

Minor Comments:

Can the authors comment more generally on the physiological need for this complex? Are these proteins organized together for the speed of responses? Specificity of responses with respect to other GPCR signalling systems? I also worry about the relevance to cardiac biology in humans where the pace of the heart is much slower- can the authors comment about translation to human cardiac biology?

2. Significance:

Significance (Required)

The findings are quite interesting, add to the existing literature regarding AC scaffolding complexes and add to our understanding as to how Gs-mediated signalling might regulate the effects of such complexes. It is a very well-written manuscript, the experiments general conducted carefully and within the small gaps noted in my comments above, it runs the gamut from in vitro to in vivo experiments that make for a compelling "translational" narrative. It will be of interest to GPCR experts like myself and more importantly to a more general readership.

3. How much time do you estimate the authors will need to complete the suggested revisions:

Estimated time to Complete Revisions (Required)

(Decision Recommendation)

Between 1 and 3 months

4. Review Commons values the work of reviewers and encourages them to get credit for their work. Select 'Yes' below to register your reviewing activity at [Publons](); note that the content of your review will not be visible on Publons.

Reviewer Publons

Yes

Review #2

1. Evidence, reproducibility and clarity:

Evidence, reproducibility and clarity (Required)

Summary:

Popeye Domain-Containing Protein 1 Scaffolds a Complex of Adenylyl Cyclase 9 and the Two-Pore-Domain Potassium Channel TREK-1 in Heart

Baldwin et al. have identified a novel Adenylyl cyclase type 9 (AC9) scaffold, the Popeye domain-containing (POPDC) protein, which binds cAMP with high affinity. They show that AC9 binds to all three isoforms of POPDC, interacting with both the transmembrane regions and the cytosolic domain of POPDC proteins by using various cellular and biochemical methods, including PLA, BiFC, FLIM-FRET, co-immunoprecipitation, followed by Adenylyl Cyclase activity and Immunoprecipitation-AC assays. Furthermore, authors could demonstrate that TREK-1 is co-localized with a complex of POPDC1/AC9 and associates with both POPDC1 and AC9 as shown by BiFC and co-immunoprecipitation assays in HEK293 cells. They claim that the binding of AC9 and POPDC is independent of cAMP production, even though AC9 association with TREK-1 is reduced in an isoproterenol (ISO)-dependent manner, requiring an intact Popeye domain and local production of cAMP within the complex. Taken together, POPDC1, therefore, represents a novel scaffolding protein for AC9 to regulate downstream effectors for heart rate control.

The work describes a new scaffolding complex involving AC9/POPDC/TREK-1 proteins in the heterologous expression systems that could certainly be of general interest to the readers, particularly to those interested in cAMP signaling, heart rate variability, and cardiac arrhythmias. Nevertheless, I have some comments:

Minor comments:

1) Table:1: row "Open Field Box Zone 1 (cm)": the difference between genotypes is rather pronounced. Is this not significantly different because there is no difference, or are the tests underpowered? i.e. was a power calculation performed in advance to the tests?

2) Mice are named differently (AC9 or Adcy9)

3) I agree with the authors that there are no good antibodies (commercial) available for AC9 and POPDC proteins for performing native co-immunoprecipitation assays to demonstrate that such a complex exists in native tissues. Growing evidence points to the fact that ion channels exist in macromolecular signaling complexes, comprising of pore-forming α-subunits, auxiliary subunits, regulatory enzymes, and proteins involved in membrane targeting. Such an approach of mass spectrometry analysis of native channel complexes has been successfully employed on TREK-1 channels (PMID: 17110924) by using brain synaptosomal proteins. It would be interesting to see whether AC9/POPDC complex could be precipitated by using TREK-1 antibodies.

4) Immunohistochemical stainings were carried out in the case of POPDC proteins (PMID: 24066022; PMID: 31551355) and for TREK-1 channels (PMID: 24101433). Did the authors try to perform IHC

experiments on native tissues?

5) Mtap2 augments TREK-1 current by recruiting the proteins to the plasma membrane (PMID: 18716213). AKAP150 modifies the gating of TREK-1 channels and thereby modifies the channel properties. It has been shown that the coexpression of both interacting proteins provides additional effects on TREK-1 current. Thus, it will be interesting to study the modulation of TREK-1 current in the presence of AC9:POPDC and these other TREK-1 interaction partners.

6) Fig 3 B: AC9 band pattern is different in the lysates obtained from sf9 cells to that of HEK cells - please discuss.

7) Fig 3D: It is unclear what authors mean with 'pC3'

8) Fig 4D: Control immunoprecipitations performed by empty GFP are missing.

2. Significance:

Significance (Required)

This work represents a novel and significant contribution to the understanding of heart rate regulation and the factors underlying these processes. However, several minor issues should be addressed before the publication of this manuscript.

3. How much time do you estimate the authors will need to complete the suggested revisions:

Estimated time to Complete Revisions (Required)

(Decision Recommendation)

Between 1 and 3 months

4. Review Commons values the work of reviewers and encourages them to get credit for their work. Select 'Yes' below to register your reviewing activity at Publons; note that the content of your review will not be visible on Publons.

Reviewer Publons

Yes

Review #3

1. Evidence, reproducibility and clarity:

Evidence, reproducibility and clarity (Required)

The study by Baldwin et al. reveals a protein complex consisting POPDC, adenylyl cyclase 9 and TREK-1 in the heart. The manuscript is we'll written and easy to follow. The data are clear and very well presented.

Minor comments:

This reviewer wonders whether it may be better to term POPDC an adaptor protein instead of terming it scaffolding protein?

The discussion is long and has sections which are redundant with other sections of the manuscript. Maybe the discussion could be shortened.

2. Significance:

Significance (Required)

The identified complex has not previously described. The finding is significant because it contributes to better understanding heart rate regulation and it may have clinical implications as a pharmacological target for interfering with arrhythmia.

3. How much time do you estimate the authors will need to complete the suggested revisions:

Estimated time to Complete Revisions (Required)

(Decision Recommendation)

Less than 1 month

4. Review Commons values the work of reviewers and encourages them to get credit for their work. Select 'Yes' below to register your reviewing activity at [Publons]; note that the content of your review will not be visible on Publons.

Reviewer Publons

Yes

Manuscript number: RC-2022-01246
Corresponding author(s): Carmen Dessauer

[The "revision plan" should delineate the revisions that authors intend to carry out in response to the points raised by the referees. It also provides the authors with the opportunity to explain their view of the paper and of the referee reports.

The document is important for the editors of affiliate journals when they make a first decision on the transferred manuscript. It will also be useful to readers of the reprint and help them to obtain a balanced view of the paper.

1. General Statements

We thank all three reviewers for recognizing the "*novel and significant contribution*" of this study, its "*translational" narrative*", the fact that "*experiments were conducted carefully*", and that "*it is a very well-written manuscript*". We were highly encouraged by the overall positive reviews and will try to accommodate reviewers suggestions, where possible. We have added most of the edits to the writing and have outlined planned experiments that are currently in progress. Location of changes specifically requested by the reviewers are marked by a black bar.

2. Description of the planned revisions

Insert here a point-by-point reply that explains what revisions, additional experimentations and analyses are planned to address the points raised by the referees.

1. As suggested by reviewer 1 (pt 2), we are adding competition experiments for the PLA analysis. Note, PLA kits are backordered until mid-April but all other reagents are on hand. Specifically, we will perform PLA between YFP-tagged AC9 and POPDC1 +/- non-tagged (or Flag-tagged) AC9, POPDC1, and EGFR (the latter serves as a negative control transmembrane protein that does not interact with POPDC1, shown in Fig 3). Several controls were already performed for BiFC in Fig. 5 between AC9 and TREK-1, including the lack BiFC signals in COS-7 cells that have greatly reduced POPDC1 and POPDC2 expression. We have now added RT-PCR of POPDC1 and POPDC2 from HEK293 and COS-7 cells in the supplemental data to show >10-fold less POPDC1/2 mRNA in COS-7 cells as compared to HEK293 cells. We have also added to the supplemental data the western blots to show equal expression of VN- and VC-tagged proteins upon isoproterenol treatments for Fig 5.

2. Reviewer 1 (pt 4) recognizes the lack of good antibodies but asked, *"Could they express single tagged versions of these proteins in cardiomyocytes and pull down the tag combined with M/S analysis to show that the other proteins are associated in cells other than HEK 293 cells or other heterologous systems?"*. We have already shown association of the endogenous proteins

in heart, but will try a few alternative strategies to address the reviewers suggestion. First, we have added the western blots for TREK-1 in the immunoprecipitations from heart in Fig 6. Second, we will try and perform M/S on endogenous TREK-1 pull-downs from heart. The only caveat is the difficulty at times of detecting low levels of transmembrane, hydrophobic proteins by mass spectrometry (AC9 represents less than 3% of total AC activity in heart). Third, we will attempt PLA in cardiomyocytes using an antibody that recognizes endogenous POPDC2 +/- expression of Flag-tagged AC9. A POPDC1 antibody is not available but since these proteins can heterodimerize (manuscript in preparation), we hope that this approach will be successful. The issue with overexpression as the reviewer suggests is that expression of only POPDC1 in the absence of POPDC2 in cardiomyocytes tends to accumulate in the ER and does not get properly localized, while expression of Flag-AC9 at higher levels necessary for M/S tends to be toxic to cardiomyocytes. Note, we would argue that the requirement for POPDC1 for interactions between AC9 and TREK-1 in heart tissue is already strong evidence for an endogenous complex of all three proteins (Fig 6).

3. Reviewer 1 (pt 5) asked, *"In Figure 6, is there some way to assess the effect isoproterenol or FSK stimulation on the amount of TREK pulled down?"*. We will try to pull-down TREK-1 protein from mice that have been injected +/- isoproterenol and determine the amount of AC9 pulled down (using activity assays) to address reviewer 1's question (pt 5). The reviewer is likely referring to only a cellular assay which will be our backup plan if the timing of the in vivo isoproterenol injection proves too tricky. Note, the validation of the TREK-1 antibody for immunoprecipitation now appears in the supplemental data.

4. An important experiment is the assessment of TREK-1 channel activity in the presence of AC9 (requested by both reviewers 1 and 2). We have gained the help of Niels Decher's lab who will perform this in Xenopus oocytes (the best model system for assessing TREK-1 regulation). His lab performed the initial experiments showing the regulation of TREK-1 by POPDC1. Oocytes will be injected with cRNA transcripts for TREK-1 and POPDC1 +/- AC9. Increased basal cAMP upon AC9 addition may be sufficient to reverse POPDC1 effects on TREK-1. If not, we will stimulate AC activity with a novel partial agonist that selectively stimulates AC9 approximately 2-fold, as was recently reported by our lab (Qi et al. Nature Commun, 2022). Note, forskolin is a poor activator of AC9 under most conditions.

5. Reviewer 2 (pt 8) requested control immunoprecipitations using empty GFP. We are adding the requested controls for figure 4D to the supplemental data (will be Fig S1).

6. Once experiments are complete we will finalize edits to the discussion. As requested by reviewer 1 (pt 7), we will address a potential role for PKA, and other Gs-coupled or Gi-coupled receptors (note, AC9 is not inhibited by Gi) in the discussion and will emphasize the need for such a complex in human heart (reviewer 1 minor comments).

3. Description of the revisions that have already been incorporated in the transferred manuscript

Please insert a point-by-point reply describing the revisions that were already carried out and included in the transferred manuscript. If no revisions have been carried out yet, please leave this section empty.

1. Three supplemental figures were added. We felt these would enhance the overall rigor of the manuscript and partially address reviewers concerns, namely a) RT-PCR for POPDC1 and POPDC2 in HEK392 versus COS-7 cells (Table S1); b) western blot of BiFC experiments showing similar expression levels of VN- and VC-tagged proteins +/- isoproterenol treatment (Fig S2); and c) validation of the TREK-1 antibody for immunoprecipitation of TREK-1 (in response to reviewer 1, pt 4,5; Fig S3). In addition, we added the western blots of TREK-1 to Figure 6.

2. As requested by reviewer 1 (pt 1), we have better explained the cardiac analysis, including defining RR interval (bottom of page 4) and methods associated with heart rate variability (RMSSD and pNN6; top of page 5).

3. As requested by reviewer 1 (pt 2), we have added language to the results (page 5) to describe functional assays of the tagged proteins (used in multiple publications now).

4. We elaborated on the difference between POPDC1 and POPDC2 in various assays probing interactions with AC9 in the results section (as requested by reviewer 1, pts 2 and 3). We added on page 6: *"POPDC2:AC9 may represent a different conformation than POPDC1:AC9, consistent with the decreased BiFC signal and AC9 activity."* Some of these differences may also be a result of heterodimerization between the POPDC isoforms (in preparation) and the reduced trafficking of POPDC2 to the plasma membrane in the absence of POPDC1. This latter point was already included in the discussion, *"POPDC2 may play a second, inhibitory role for AC9. Alternatively, given the possible existence of heteromeric complexes consisting of POPDC1 and POPDC2, fully active AC9 may bind to POPDC1 homodimers and possibly a POPDC1/2 heteromeric complex, while an AC9 enzyme incapable of Gαs stimulation is associated with POPDC2. The complex regulation of AC activity by POPDC proteins remains under investigation."*

5. We added information about sample size calculations for heart rate measurements to the statistical analysis section of the methods to address a minor comment (reviewer 2, pt 1). Note, we did not perform power analysis for the open field test per se since we did not anticipate a difference and we were using these behavioral assays to control for issues related to the telemetry probe implants. The large variability between animals of even the same genotype and smaller group sizes may have resulted in the lack of a statistically significant result. However, it did not make sense to us why in a running wheel, *Adcy9*$^{-/-}$ ran less than WT (P=0.2), but in the open field box, the total distance traveled by *Adcy9*$^{-/-}$ was actually greater than WT after probe

implant (P=0.48). Moreover, we also performed open field box tests prior to surgery on WT and *Adcy9*$^{-/-}$ (n=8 for both; not reported) but in this case WT entered zone 1 more often before surgery (P=0.09), while *Adcy9*$^{-/-}$ entered zone 1 more often post-surgery (P=0.3). It is possible that WT are simply not as curious of the box the second time around. Given all of this, we are inclined to believe there isn't a significant difference in mobility or anxiety.

6. We have ensured that all references to *Adcy9*$^{-/-}$ mice use consistent nomenclature (reviewer 2, pt 2).

7. We added clarifications of different AC9 bands (due to glycosylation) in the legend for Figure 3 (the first place this appears). We changed pC3 to indicate empty vector control in figure 3 (to address pts 6 and 7 of reviewer 2).

8. We have altered the description of POPDC1 in the introduction and discussion as an adaptor for this particular complex, as suggested by reviewer 3. However, we have left open the possibility that POPDC may also bring PDE and other regulatory molecules to the complex, thus acting more as a scaffold.

9. We have reduced the discussion somewhat as suggested by reviewer 3. Further edits will be made to the discussion once additional experiments are added.

4. Description of analyses that authors prefer not to carry out

Please include a point-by-point response explaining why some of the requested data or additional analyses might not be necessary or cannot be provided within the scope of a revision. This can be due to time or resource limitations or in case of disagreement about the necessity of such additional data given the scope of the study. Please leave empty if not applicable.

1. We agree with reviewer 2 that understanding how AKAP79 and Mtap2 regulation combines with that of POPDC1 will be of high interest, but believe the complexity added by these additional regulators should be studied in a subsequent study.

2. Immunohistochemical stainings of POPDC proteins and TREK-1 have been previously published as correctly noted by reviewer 2, pt 4. Immunohistochemistry of endogenous AC9 is sadly not possible due to lack of available antibodies and the low level of expression. However, we have previously published immunocytochemistry of Flag-tagged AC9 expressed in neonatal cardiomyocytes (Li et al. Cells 2019). All three proteins localize to membranes of cardiomyocytes, with AC9 found in particular at the intercalated discs, where POPDC (reviewed by Gruscheski and Brand, 2021) and TREK-1 and bIV-spectrin (Hund TJ, 2014) are localized. This has now been added to the discussion.

1st Editorial Decision 13th Apr 2022

Dear Dr. Dessauer,

Thank you for transferring your manuscript to EMBO Reports, which was previously reviewed at Review Commons.

Having looked at all documents including your revision plan, we would like to invite you to revise your manuscript with the understanding that the referee concerns (as in their reports) must be fully addressed and their suggestions taken on board. Please address all referee concerns in a complete point-by-point response. Acceptance of the manuscript will depend on a positive outcome of a second round of review. It is EMBO reports policy to allow a single round of revision only and acceptance or rejection of the manuscript will therefore depend on the completeness of your responses included in the next, final version of the manuscript.

We generally allow three months as standard revision time. As a matter of policy, competing manuscripts published during this period will not negatively impact on our assessment of the conceptual advance presented by your study. However, we request that you contact the editor as soon as possible upon publication of any related work, to discuss how to proceed. Should you foresee a problem in meeting this three-month deadline, please let us know in advance and we may be able to grant an extension.

*** Temporary update to EMBO Press scooping protection policy:
We are aware that many laboratories cannot function at full efficiency during the current COVID-19/SARS-CoV-2 pandemic and have therefore extended our 'scooping protection policy' to cover the period required for a full revision to address the experimental issues highlighted in the editorial decision letter. Please contact the scientific editor handling your manuscript to discuss a revision plan should you need additional time, and also if you see a paper with related content published elsewhere.***

IMPORTANT NOTE: we perform an initial quality control of all revised manuscripts before re-review. Your manuscript will FAIL this control and the handling will be DELAYED if the following APPLIES:
1. A data availability section providing access to data deposited in public databases is missing (where applicable).
2. Your manuscript contains statistics and error bars based on n=2. Please use scatter plots in these cases.

You can submit the revision either as a Scientific Report or as a Research Article. For Scientific Reports, the revised manuscript can contain up to 5 main figures and 5 Expanded View figures. If the revision leads to a manuscript with more than 5 main figures it will be published as a Research Article. In this case the Results and Discussion section should be separate. If a Scientific Report is submitted, these sections have to be combined. This will help to shorten the manuscript text by eliminating some redundancy that is inevitable when discussing the same experiments twice. In either case, all materials and methods should be included in the main manuscript file

Supplementary/additional data: The Expanded View format, which will be displayed in the main HTML of the paper in a collapsible format, has replaced the Supplementary information. You can submit up to 5 images as Expanded View. Please follow the nomenclature Figure EV1, Figure EV2 etc. The figure legend for these should be included in the main manuscript document file in a section called Expanded View Figure Legends after the main Figure Legends section. Additional Supplementary material should be supplied as a single pdf labeled Appendix. The Appendix includes a table of content on the first page with page numbers, all figures and their legends. Please follow the nomenclature Appendix Figure Sx throughout the text and also label the figures according to this nomenclature. For more details please refer to our guide to authors.

Please note that for all articles published beginning 1 July 2020, the EMBO Reports reference style will change to the Harvard style for all article types. Details and examples are provided at
https://www.embopress.org/page/journal/14693178/authorguide#referencesformat

When submitting your revised manuscript, please carefully review the instructions that follow below. Failure to include requested items will delay the evaluation of your revision.

1) a .docx formatted version of the manuscript text (including legends for main figures, EV figures and tables). Please make sure that the changes are highlighted to be clearly visible.

2) individual production quality figure files as .eps, .tif, .jpg (one file per figure).

3) a .docx formatted letter INCLUDING the reviewers' reports and your detailed point-by-point responses to their comments. As part of the EMBO Press transparent editorial process, the point-by-point response is part of the Review Process File (RPF), which will be published alongside your paper. For more details on our Transparent Editorial Process, please visit our website:
https://www.embopress.org/page/journal/14693178/authorguide#transparentprocess
You are able to opt out of this by letting the editorial office know (emboreports@embo.org). If you do opt out, the Review Process File link will point to the following statement: "No Review Process File is available with this article, as the authors have chosen not to make the review process public in this case."

4) a complete author checklist, which you can download from our author guidelines (). Please insert information in the checklist that is also reflected in the manuscript. The completed author checklist will also be part of the RPF.

5) Please note that all corresponding authors are required to supply an ORCID ID for their name upon submission of a revised manuscript (). Please find instructions on how to link your ORCID ID to your account in our manuscript tracking system in our Author guidelines ().

6) We replaced Supplementary Information with Expanded View (EV) Figures and Tables that are collapsible/expandable online. A maximum of 5 EV Figures can be typeset. EV Figures should be cited as 'Figure EV1, Figure EV2" etc... in the text and their respective legends should be included in the main text after the legends of regular figures.

- For the figures that you do NOT wish to display as Expanded View figures, they should be bundled together with their legends in a single PDF file called *Appendix*, which should start with a short Table of Content. Appendix figures should be referred to in the main text as: "Appendix Figure S1, Appendix Figure S2" etc. See detailed instructions regarding expanded view here: .

- Additional Tables/Datasets should be labeled and referred to as Table EV1, Dataset EV1, etc. Legends have to be provided in a separate tab in case of .xls files. Alternatively, the legend can be supplied as a separate text file (README) and zipped together with the Table/Dataset file.

7) We would also encourage you to include the source data for figure panels that show essential data.

Numerical data should be provided as individual .xls or .csv files (including a tab describing the data). For blots or microscopy, uncropped images should be submitted (using a zip archive if multiple images need to be supplied for one panel). Additional information on source data and instruction on how to label the files are available .

8) Our journal encourages inclusion of *data citations in the reference list* to directly cite datasets that were re-used and obtained from public databases. Data citations in the article text are distinct from normal bibliographical citations and should directly link to the database records from which the data can be accessed. In the main text, data citations are formatted as follows: "Data ref: Smith et al, 2001" or "Data ref: NCBI Sequence Read Archive PRJNA342805, 2017". In the Reference list, data citations must be labeled with "[DATASET]". A data reference must provide the database name, accession number/identifiers and a resolvable link to the landing page from which the data can be accessed at the end of the reference. Further instructions are available at .

9) Please make sure to include a Data Availability Section before submitting your revision - if it is not applicable, make a statement that no data were deposited in a public database. Primary datasets (and computer code, where appropriate) produced in this study need to be deposited in an appropriate public database (see).

Please remember to provide a reviewer password if the datasets are not yet public.

The accession numbers and database should be listed in a formal "Data Availability " section (placed after Materials & Method) that follows the model below. Please note that the Data Availability Section is restricted to new primary data that are part of this study.

Data availability

The datasets (and computer code) produced in this study are available in the following databases:

- RNA-Seq data: Gene Expression Omnibus GSE46843 (https://www.ncbi.nlm.nih.gov/geo/query/acc.cgi?acc=GSE46843)
- [data type]: [name of the resource] [accession number/identifier/doi] ([URL or identifiers.org/DATABASE:ACCESSION])

*** Note - All links should resolve to a page where the data can be accessed. ***

10) Regarding data quantification, please ensure to specify the name of the statistical test used to generate error bars and P values, the number (n) of independent experiments underlying each data point (not replicate measures of one sample), and the test used to calculate p-values in each figure legend. Discussion of statistical methodology can be reported in the materials and methods section, but figure legends should contain a basic description of n, P and the test applied.
Please note that error bars and statistical comparisons may only be applied to data obtained from at least three independent biological replicates.
Please also include scale bars in all microscopy images.

We would also welcome the submission of cover suggestions, or motifs to be used by our Graphics Illustrator in designing a cover.

11) The journal requires a statement specifying whether or not authors have competing interests (defined as all potential or actual interests that could be perceived to influence the presentation or interpretation of an article). In case of competing interests, this must be specified in your disclosure statement. Further information: https://www.embopress.org/competing-interests

I look forward to seeing a revised version of your manuscript when it is ready. Please let me know if you have questions or comments regarding the revision.

Kind regards,

Deniz Senyilmaz Tiebe

Deniz Senyilmaz Tiebe, PhD
Editor
EMBO Reports

Popeye Domain-Containing Protein 1 Scaffolds a Complex of Adenylyl Cyclase 9 and the Two-Pore-Domain Potassium Channel TREK-1 in Heart

Tanya A. Baldwin[1†], Yong Li[1†], Autumn Marsden[1], Susanne Rinné[4], Anibal Garza Carbajal[1], Roland F.R. Schindler[3], Musi Zhang[1], Mia A. Garcia[1], Venugopal Reddy Venna[2], Niels Decher[4], Thomas Brand[3], and Carmen W. Dessauer[1*]

Author's Point-by-point Response Letter for the reviewer's reports.
(Author's responses are in blue)

Reviewer #1 (Evidence, reproducibility and clarity (Required)):

Summary: The authors have a novel adenylyl cyclase (AC) scaffold, the Popeye domain-containing (POPDC) protein. They demonstrate that unlike other currently known AC scaffolding proteins such as AKAPs, POPDC1 binds cAMP with high affinity. As the POPDC family of proteins have been implicated in cardiac pacemaking and conduction, in part because of cAMP-dependent regulation of TREK-1 potassium channels. The authors next show that TREK-1 binds the AC9:POPDC1 complex and co-purifies with AC9-associated activity in heart in way that depends on the presence of POPDC1. They note that the interaction of AC9 and POPDC1 was cAMP independent. However, TREK-1 association with AC9/POPDC1 was reduced in an isoproterenol-dependent manner, requiring both an intact cAMP binding Popeye domain and AC activity in the complex. Finally loss of AC9 in a transgenic mouse model led to bradycardia at rest and isoproterenol-induced heart rate variability, a phenotype previously seen following loss of Popdc1, and similar to loss of TREK-1.

Major Comments:

1) The authors start, convincingly with in vivo experiments. I feel that for the non-specialist reader, the parameters associated with their analysis, RR interval and methods such as RMSSD and pNN6 could be better explained.

Thank you. Full definitions are now included in the results.

2) PLA and BiFC experiments in Figure 2. It is good that the authors used multiple techniques to demonstrate the interaction and in general I acknowledge that a paucity of good antibodies is a legitimate technical issue in studies such as this. However, some additional controls might be suggested that involve competition of the PLA or BiFC signals by untagged competitor proteins. Also, are the proteins tagged still functional? The results shown with different POPDCs are not entirely consistent between techniques- is this related to loss of functionality in some sense?

We have added references to address the functionality of the tagged proteins in the results. We also added competition experiments for the PLA analysis in HEK293 cells (see new Fig EV1C). Specifically, we performed PLA between YFP-tagged AC9 and Myc-tagged POPDC1 +/- non-tagged (or Flag-tagged) AC9, POPDC1, and EGFR (the latter serves as a negative control transmembrane protein that does not interact with POPDC1, as shown in Fig 3). Several controls were already performed for BiFC in Fig. 5 between AC9 and TREK-1, including the lack of BiFC signals in COS-7 cells that have greatly reduced POPDC1 and POPDC2 expression. We have now added RT-PCR of POPDC1 and POPDC2 from HEK293 and COS-7 cells in the supplemental data to show >10-fold less POPDC1/2 mRNA in COS-7 cells as compared to HEK293 cells. We have also added to the supplemental data the western blots to show equal expression of VN- and VC-tagged proteins upon isoproterenol treatments for Fig 5.

The only major difference between techniques for POPDC proteins is with regards to POPDC2. It has a reduced or absent response in cellular assays (BiFC in HEK293 cells and PLA in neonatal CMs, respectively) but can clearly interact by immunoprecipitation when overexpressed. Some of these differences may simply be due to the distance constraints of BiFC and PLA techniques. The surprising result was the lack of AC activity upon pull-down of POPDC2. This is now addressed more fully in the results and discussion. POPDC2 can heterodimerize with POPDC1 (paper in preparation) and the varying presence of POPDC1 may account for some of these differences with respect to cellular assays.

3) In the IP-AC assays, POPDC1 and POPDC2 have differential responses to Gs stimulation yet both still are associated with the complex. What is the significance of this?

As stated above, POPDC1 and POPDC2 can form heteromers. Thus, fully active AC9 may bind to POPDC1 homodimers and possibly a POPDC1/2 heteromeric complex, while an AC9 enzyme incapable of Gαs stimulation is associated with POPDC2. It is unclear if the differential responses are due to detergents associated with the IP of POPDC2 (AC9 is normally fully active under these conditions), or if this is pointing towards a form of AC regulation. The complex regulation of AC activity by POPDC proteins remains under investigation and is poorly understood at this time. The following appears in the discussion. *"POPDC2 may play a second, inhibitory role for AC9. Alternatively, given the possible existence of heteromeric complexes consisting of POPDC1 and POPDC2, fully active AC9 may bind to POPDC1 homodimers and possibly a POPDC1/2 heteromeric complex, while an AC9 enzyme incapable of Gαs stimulation is associated with POPDC2. The complex regulation of AC activity by POPDC proteins remains under investigation."*

4) With regard to demonstrating the interaction in homologous systems, the authors rightly bring up the absence of good antibodies. Could they express single tagged versions of these proteins in cardiomyocytes and pull down the tag combined with M/S analysis to show that the other proteins are associated in cells other than HEK 293 cells or other heterologous systems?

To complement our other cellular interaction studies using cardiomyocytes, we have expressed Flag-tagged GFP versus Flag-tagged AC9 in neonatal cardiomyocytes and performed proximity ligation assay with antibodies against FLAG and the endogenous POPDC1 and POPDC2 proteins, using Gβγ as a positive control (new Fig 2B,C). POPDC1 and POPDC2 antibodies have been previously validated for immunohistochemistry (although they do not work well for IP or WB of endogenous proteins). POPDC1 but not POPDC2 produced a positive PLA signal. We preferred this method due to the difficulty of detecting low levels of transmembrane, hydrophobic proteins by mass spectrometry (AC9 represents less than 3% of total AC activity in heart). Note, we would argue that the requirement for POPDC1 for interactions between AC9 and TREK-1 in heart tissue is already strong evidence for an endogenous complex of all three proteins (Fig 5).

5) In Figure 6, is there some way to assess the effect isoproterenol or FSK stimulation on the amount of TREK pulled down?

Yes! A new experiment performed in HEK293 cells relies on pull-down of TREK-1 protein (using the TREK-1 antibody) with AC9 and POPDC1 in the absence or presence of isoproterenol (10 min treatment). There is a similar ~40% decrease in AC9 and POPDC1 binding to TREK-1 after isoproterenol treatment (new Fig 6) as seen with BiFC. Note, the validation of the TREK-1 antibody for immunoprecipitation now appears in the supplemental data.

6) Is there some way to assess the effect of this complex on TREK channel activity in a heterologous system?

We have gained the help of Niels Decher's lab who has measured TREK-1 currents in Xenopus oocytes (the best model system for assessing TREK-1 regulation). His lab performed the initial experiments showing the enhanced trafficking of TREK-1 to the PM and the increase in TREK-1 currents by POPDC1. Oocytes were injected with cRNA transcripts for TREK-1 and POPDC1 +/- AC9. Given the multiple mechanisms of TREK-1 regulation by cAMP, the effects are complex. Therefore we also compared as controls the catalytically inactive AC9D and theophylline-treated conditions to increase cAMP. When normalized to effects of TREK-1 in the presence of POPDC1, AC9 and AC9D show opposing effects on TREK-1 currents, particularly in the presence of theophylline. Importantly, AC9D can protect POPDC1 from theophylline treatment, allowing enhancement of TREK-1 currents. These exciting new results are shown in Fig 8.

7) Might we expect that PKA is involved here as well or does cAMP binding act as the primary determinant of complex dissocation? Is there a concomitant role for Gi-coupled receptors in upregulating the activity of the complex? Are the effects they measured phenocopied by other Gs-coupled receptors or direct activation of AC with forskolin?

Previous studies show that cAMP binding to POPDC1 is the primary determinant of TREK-1 association with POPDC1 (Froese et al, 2012). However, we cannot rule out that both modes of regulation are present and active in cells where AKAP79 is also expressed. We have added a new section to the discussion to address the role of Gi-coupled receptors for AKAP79 versus POPDC1 scaffolded TREK-1 complexes (see below). Note, forskolin is a poor activator of AC9 under most conditions while Gαi does not directly inhibit AC9, thus the regulation of a complex containing AC9 will be quite different than those anchored by AKAPs with other cardiac AC isoforms.

Minor Comments: Can the authors comment more generally on the physiological need for this complex? Are these proteins organized together for the speed of responses? Specificity of responses with respect to other GPCR signalling systems? I also worry about the relevance to cardiac biology in humans where the pace of the heart is much slower- can the authors comment about translation to human cardiac biology?

To further elaborate on the need/function of this complex (and address point 7 as well), the following paragraph was added to the discussion. *"This model suggests differential regulation of TREK-1 by AKAP79- versus POPDC1-bound ACs. First, TREK-1 inhibition by PKA on AKAP79 (Noël et al., 2011) is likely regulated by additional feedback inhibition of bound AC5, AC6, and AC8 by PKA (Baldwin & Dessauer, 2018; Marsden & Dessauer, 2019; Musheshe et al., 2018), while PKA regulation of AC9 within a AC9:POPDC1:TREK-1 complex has not been reported (Baldwin et al., 2019; Kawaguchi et al., 2008; Lolicato et al., 2017)). Second, while nearly all the AKAP79-bound cardiac ACs are stimulated by forskolin and inhibited by Gαi, AC9 is insensitive to these regulators (Baldwin et al., 2019; Kawaguchi et al., 2008; Lolicato et al., 2017)), suggesting that the AC9:POPDC1:TREK-1 complex is not regulated by Gi-coupled receptors. Third, if association of Ca^{2+}-stimulated AC activity with TREK-1 in heart is due to AC8:AKAP79 scaffolding, then inhibition of TREK-1 within AKAP, but not POPDC1, complexes would be Ca^{2+}-sensitive. Finally, the nanometer size of cAMP domains and the failure of these domains to cross-talk with other GPCRs at low agonist concentrations suggests that AKAP and POPDC1 molecular complexes dictate the specificity of cellular responses regulating TREK-1 (Anton et al, 2022)."*

In terms of the relevance to human cardiac biology, we argued the following in the discussion. *"The functional conservation of cardiac arrhythmia phenotypes of POPDC1 mutations from zebrafish, mice and humans suggests that the identified POPDC1 complexes are likely relevant in regulating cardiac function in the human heart as well."*

Reviewer #1 (Significance (Required)):

The findings are quite interesting, add to the existing literature regarding AC scaffolding complexes and add to our understanding as to how Gs-mediated signalling might regulate the effects of such complexes. It is a very well-written manuscript, the experiments general conducted carefully and within the small gaps noted in my comments above, it runs the gamut from in vitro to in vivo experiments that make for a compelling "translational" narrative. It will be of interest to GPCR experts like myself and more importantly to a more general readership.

Reviewer #2 (Evidence, reproducibility and clarity (Required)):

Summary:
Popeye Domain-Containing Protein 1 Scaffolds a Complex of Adenylyl Cyclase 9 and the Two-Pore-Domain Potassium Channel TREK-1 in Heart

Baldwin et al. have identified a novel Adenylyl cyclase type 9 (AC9) scaffold, the Popeye domain-containing (POPDC) protein, which binds cAMP with high affinity. They show that AC9 binds to all three isoforms of POPDC, interacting with both the transmembrane regions and the cytosolic domain of POPDC proteins by using various cellular and biochemical methods, including PLA, BiFC, FLIM-FRET, co-immunoprecipitation, followed by Adenylyl Cyclase activity and Immunoprecipitation-AC assays. Furthermore, authors could demonstrate that TREK-1 is co-localized with a complex of POPDC1/AC9 and associates with both POPDC1 and AC9 as shown by BiFC and co-immunoprecipitation assays in HEK293 cells. They claim that the binding of AC9 and POPDC is independent of cAMP production, even though AC9 association with TREK-1 is reduced in an isoproterenol (ISO)-dependent manner, requiring an intact Popeye domain and local production of cAMP within the complex. Taken together, POPDC1, therefore, represents a novel scaffolding protein for AC9 to regulate downstream effectors for heart rate control. The work describes a new scaffolding complex involving AC9/POPDC/TREK-1 proteins in the heterologous expression systems that could certainly be of general interest to the readers, particularly to those interested in cAMP signaling, heart rate variability, and cardiac arrhythmias. Nevertheless, I have some comments:

Minor comments:
1, Table:1: row "Open Field Box Zone 1 (cm)": the difference between genotypes is rather pronounced. Is this not significantly different because there is no difference, or are the tests underpowered? i.e. was a power calculation performed in advance to the tests?

We added information about sample size calculations for heart rate measurements to the statistical analysis section of the methods. Note, we did not perform power analysis for the open field test per se since we did not anticipate a difference and we were using these behavioral assays to control for issues related to the telemetry probe implants. The large variability between animals of even the same genotype and smaller group sizes may have resulted in the lack of a statistically significant result. However, it is unclear why in a running wheel, *Adcy9*$^{-/-}$ ran less than WT (P=0.2), but in the open field box, the total distance traveled by *Adcy9*$^{-/-}$ was actually greater than WT after probe implant (P=0.48). Moreover, we also performed open field box tests prior to surgery on WT and *Adcy9*$^{-/-}$ (n=8 for both; not reported) but in this case WT entered zone 1 more often before surgery (P=0.09), while *Adcy9*$^{-/-}$ entered zone 1 more often post-surgery (P=0.3). It is possible that WT are simply not as curious of the box the second time around. Given all of this, we are inclined to believe there isn't a significant difference in mobility or anxiety.

2, Mice are named differently (AC9 or Adcy9) – Thank you. We have ensured that all references to *Adcy9*$^{-/-}$ mice use consistent nomenclature.

3, I agree with the authors that there are no good antibodies (commercial) available for AC9 and POPDC proteins for performing native co-immunoprecipitation assays to demonstrate that such a complex exists in native tissues. Growing evidence points to the fact that ion channels exist in macromolecular signaling complexes, comprising of pore-forming α-subunits, auxiliary subunits, regulatory enzymes, and proteins involved in membrane targeting. Such an approach of mass spectrometry analysis of native channel complexes has been successfully employed on TREK-1 channels (PMID: 17110924) by using brain synaptosomal proteins. It would be interesting to see whether AC9/POPDC complex could be precipitated by using TREK-1 antibodies.

We agree that understanding how AKAP79 and Mtap2 regulation combines with that of POPDC1 will be of high interest, but believe the complexity added by these additional regulators should be studied in detail in a subsequent study.

4, Immunohistochemical stainings were carried out in the case of POPDC proteins (PMID: 24066022; PMID: 31551355) and for TREK-1 channels (PMID: 24101433). Did the authors try to perform IHC experiments on native tissues?

Immunohistochemical stainings of POPDC proteins and TREK-1 have been previously published as correctly noted by the reviewer. Immunohistochemistry of endogenous AC9 is sadly not possible due to lack of available antibodies and the low level of expression. However, we have previously published immunocytochemistry of Flag-tagged AC9 expressed in neonatal cardiomyocytes (Li et al. Cells 2019). TREK-1, POPDC1, and AC9 all localize to membranes of cardiomyocytes, with AC9 found in particular at the intercalated discs, where POPDC (reviewed by Gruscheski and Brand, 2021) and TREK-1 and bIV-spectrin (Hund TJ, 2014) are localized. This has now been added to the discussion.

5, Mtap2 augments TREK-1 current by recruiting the proteins to the plasma membrane (PMID: 18716213). AKAP150 modifies the gating of TREK-1 channels and thereby modifies the channel properties. It has been shown that the coexpression of both interacting proteins provides additional effects on TREK-1 current. Thus, it will be interesting to study the modulation of TREK-1 current in the presence of AC9:POPDC and these other TREK-1 interaction partners.

As noted in response to reviewer 1, point 6, we recruited Dr. Niels Decher to measure TREK-1 currents in the presence of POPDC1 and AC9. We agree with reviewer 2 that understanding how AKAP79 and Mtap2 regulation combines with that of POPDC1 will be of high interest, but believe the complexity added by these additional regulators should be studied in a subsequent study.

6, Fig 3 B: AC9 band pattern is different in the lysates obtained from sf9 cells to that of HEK cells - please discuss.

Differences in glycosylation is often seen for AC isoforms in different cell lines. We added clarifications of the presence of multiple AC9 bands (due to glycosylation and dimerization) in the methods for immunoprecipitations and western blots.

7, Fig 3D: It is unclear what authors mean with 'pC3'

Thank you. We changed pC3 to indicate empty vector control in figure 3

8, Fig 4D: Control immunoprecipitations performed by empty GFP are missing.

These controls now appear in Appendix Fig 1.

Reviewer #2 (Significance (Required)):

This work represents a novel and significant contribution to the understanding of heart rate regulation and the factors underlying these processes. However, several minor issues should be addressed before the publication of this manuscript.

Reviewer #3 (Evidence, reproducibility and clarity (Required)):

The study by Baldwin et al. reveals a protein complex consisting POPDC, adenylyl cyclase 9 and TREK-1 in the heart. The manuscript is we'll written and easy to follow. The data are clear and very well presented.

Minor:
This reviewer wonders whether it may be better to term POPDC an adaptor protein instead of terming it scaffolding protein?

Possibly, but "*recently, an interaction of POPDC1 and PDE4 isoforms has been described (Tibbo et al, 2020). A cell-permeable peptide that disrupts the POPDC1:PDE4 complex caused a reduction in cycle length of spontaneous Ca^{2+} transients in mouse SA nodes. This effect of the peptide was only seen at baseline and was blunted after ISO stimulation.*" Thus POPDC1 may be acting as a true scaffold if both AC and TREK are also associated with a POPDC1:PDE4 complex. We have altered the wording to use adaptor when discussion of just TREK-1:POPDC1:AC9 but use the term scaffold when discussing more general effects of POPDC1.

The discussion is long and has sections which are redundant with other sections of the manuscript. Maybe the discussion could be shortened.

We tried to trim down the discussion as much as possible, while adding verbiage requested by reviewers 1 and 2 and discussion of new data for TREK-1 currents. We also removed redundant materials found in the results. Thank you.

Reviewer #3 (Significance (Required)):

The identified complex has not previously described. The finding is significant because it contributes to better understanding heart rate regulation and it may have clinical implications as a pharmacological target for interfering with arrhythmia.

1st Revision - Editorial Decision 20th Sep 2022

Dear Carmen,

Thank you for the submission of your revised manuscript to EMBO reports. I had already informed you about the positive evaluation by the two referees who both support publication.

Before we can proceed with the official acceptance, I need you to address some minor editorial points below:

- Please reduce the number of keywords to 5.

- Please update the 'Conflict of interest' paragraph to our new 'Disclosure and competing interests statement'. For more information see
https://www.embopress.org/page/journal/14693178/authorguide#conflictsofinterest

- Regarding the Author Contributions, we now use CRediT to specify the contributions of each author in the journal submission system. CRediT replaces the author contribution section. Please remove the author contributions from the manuscript text. You can use the free text box in the system to provide more detailed descriptions. See also guide to authors
https://www.embopress.org/page/journal/14693178/authorguide#authorshipguidelines.

- Please add callouts to Figure panels 6A-C.
- We generally recommend arranging figures in a manner that allows to call out the panels in an alphabetical order. In this context we note that Fig. 8E is called out before 8C. Can the panels be swapped in the figure?

- Appendix: The names need correcting throughout to Appendix Figure S# and Appendix Table S#.

- Please remove your ORCID IDs, the two-sentence summary and the Appendix legends from the manuscript file. (But note my comment in the Appendix legends in the attached file)

- Please correct the heading "Methods" to "Materials and Methods".

- I attach to this email a related manuscript file with comments by our data editors. Please address all comments and upload a revised file with tracked changes with your final manuscript submission.

- Please rephrase the abstract to make it more accessible to non-specialist readers. I added some comments in the attached word file.

We look forward to seeing a final version of your manuscript as soon as possible.

With kind regards,

Martina

Martina Rembold, PhD
Senior Editor
EMBO reports

****

Referee #1:

The authors have provided a comprehensive response to the critiques received.

Referee #2:

The authors have adequately addressed all my questions. Therefore, I have no further comments.

The authors have addressed all minor editorial requests.

2nd Revision - Editorial Decision 29th Sep 2022

Dr. Carmen W. Dessauer
McGovern Medical School, University of Texas Health Science Center at Houston
Dept. of Integrative Biology and Pharmacology
6431 Fannin St.
Houston, TX 77030
United States

Dear Carmen,

I am very pleased to accept your manuscript for publication in the next available issue of EMBO reports. Thank you for your contribution to our journal.

At the end of this email I include important information about how to proceed. Please ensure that you take the time to read the information and complete and return the necessary forms to allow us to publish your manuscript as quickly as possible.

As part of the EMBO publication's Transparent Editorial Process, EMBO reports publishes online a Review Process File to accompany accepted manuscripts. As you are aware, this File will be published in conjunction with your paper and will include the referee reports, your point-by-point response and all pertinent correspondence relating to the manuscript.

If you do NOT want this File to be published, please inform the editorial office within 2 days, if you have not done so already, otherwise the File will be published by default [contact: emboreports@embo.org]. If you do opt out, the Review Process File link will point to the following statement: "No Review Process File is available with this article, as the authors have chosen not to make the review process public in this case."

Should you be planning a Press Release on your article, please get in contact with emboreports@wiley.com as early as possible, in order to coordinate publication and release dates.

Thank you again for your contribution to EMBO reports and congratulations on a successful publication. Please consider us again in the future for your most exciting work.

Kind regards,

Martina

Martina Rembold, PhD
Senior Editor
EMBO reports

****

THINGS TO DO NOW:

You will receive proofs by e-mail approximately 2-3 weeks after all relevant files have been sent to our Production Office; you should return your corrections within 2 days of receiving the proofs.

EMBO Press participates in many Publish and Read agreements that allow authors to publish Open Access with reduced/no publication charges. Check your eligibility: https://authorservices.wiley.com/author-resources/Journal-Authors/open-access/affiliation-policies-payments/index.html

Please inform us if there is likely to be any difficulty in reaching you at the above address at that time. Failure to meet our deadlines may result in a delay of publication, or publication without your corrections.

All further communications concerning your paper should quote reference number EMBOR-2022-55208V3 and be addressed to emboreports@wiley.com.

Should you be planning a Press Release on your article, please get in contact with emboreports@wiley.com as early as possible, in order to coordinate publication and release dates.

EMBO Press Author Checklist

Corresponding Author Name: Carmen W. Dessauer	
Journal Submitted to: EMBO Reports	
Manuscript Number: EMBOR-2022-55208V2	

USEFUL LINKS FOR COMPLETING THIS FORM
The EMBO Journal - Author Guidelines
EMBO Reports - Author Guidelines
Molecular Systems Biology - Author Guidelines
EMBO Molecular Medicine - Author Guidelines

Reporting Checklist for Life Science Articles (updated January 2022)
This checklist is adapted from Materials Design Analysis Reporting (MDAR) Checklist for Authors. MDAR establishes a minimum set of requirements in transparent reporting in the life sciences (see Statement of Task: 10.31222/osf.io/9sm4x). Please follow the journal's guidelines in preparing your manuscript.
Please note that a copy of this checklist will be published alongside your article.

Abridged guidelines for figures
1. Data
The data shown in figures should satisfy the following conditions:
- the data were obtained and processed according to the field's best practice and are presented to reflect the results of the experiments in an accurate and unbiased manner.
- ideally, figure panels should include only measurements that are directly comparable to each other and obtained with the same assay.
- plots include clearly labeled error bars for independent experiments and sample sizes. Unless justified, error bars should not be shown for technical replicates.
- if n<5, the individual data points from each experiment should be plotted. Any statistical test employed should be justified.
- Source Data should be included to report the data underlying figures according to the guidelines set out in the authorship guidelines on Data Presentation.

2. Captions
Each figure caption should contain the following information, for each panel where they are relevant:
- a specification of the experimental system investigated (eg cell line, species name).
- the assay(s) and method(s) used to carry out the reported observations and measurements.
- an explicit mention of the biological and chemical entity(ies) that are being measured.
- an explicit mention of the biological and chemical entity(ies) that are altered/varied/perturbed in a controlled manner.
- the exact sample size (n) for each experimental group/condition, given as a number, not a range;
- a description of the sample collection allowing the reader to understand whether the samples represent technical or biological replicates (including how many animals, litters, cultures, etc.).
- a statement of how many times the experiment shown was independently replicated in the laboratory.
- definitions of statistical methods and measures:
  - common tests, such as t-test (please specify whether paired vs. unpaired), simple χ2 tests, Wilcoxon and Mann-Whitney tests, can be unambiguously identified by name only, but more complex techniques should be described in the methods section;
  - are tests one-sided or two-sided?
  - are there adjustments for multiple comparisons?
  - exact statistical test results, e.g., P values = x but not P values < x;
  - definition of 'center values' as median or average;
  - definition of error bars as s.d. or s.e.m.

Please complete ALL of the questions below.
Select "Not Applicable" only when the requested information is not relevant for your study.

Materials

Newly Created Materials	Information included in the manuscript?	In which section is the information available? (Reagents and Tools Table, Materials and Methods, Figures, Data Availability Section)
New materials and reagents need to be available; do any restrictions apply?	Yes	New plasmids are described in Materials and Method; no restrictions apply.

Antibodies	Information included in the manuscript?	In which section is the information available? (Reagents and Tools Table, Materials and Methods, Figures, Data Availability Section)
For **antibodies** provide the following information:<br>- Commercial antibodies: RRID (if possible) or supplier name, catalogue number and or/clone number<br>- Non-commercial: RRID or citation	Yes	Materials and Methods, under Proximity Ligation assay and Immunoprecipitations and Western Blotting.

DNA and RNA sequences	Information included in the manuscript?	In which section is the information available? (Reagents and Tools Table, Materials and Methods, Figures, Data Availability Section)
**Short novel DNA or RNA including primers, probes**: provide the sequences.	Yes	Under Appendix, Table I

Cell materials	Information included in the manuscript?	In which section is the information available? (Reagents and Tools Table, Materials and Methods, Figures, Data Availability Section)
**Cell lines:** Provide species information, strain. Provide accession number in repository **OR** supplier name, catalog number, clone number, and/**OR** RRID.	Yes	Materials and methods; Cell culture and transfections.
**Primary cultures:** Provide species, strain, sex of origin, genetic modification status.	Yes	Materials and methods; Cell culture and transfections.
Report if the cell lines were recently **authenticated** (e.g., by STR profiling) and tested for mycoplasma contamination.	Yes	Materials and methods; Cell culture and transfections.

Experimental animals	Information included in the manuscript?	In which section is the information available? (Reagents and Tools Table, Materials and Methods, Figures, Data Availability Section)
**Laboratory animals or Model organisms:** Provide species, strain, sex, age, genetic modification status. Provide accession number in repository **OR** supplier name, catalog number, clone number, **OR** RRID.	Yes	Material and methods; Gene-Targeted Mice, Voltage clamp measurement of TREK-1 current
**Animal observed in or captured from the field:** Provide species, sex, and age where possible**.**	Not Applicable	
Please detail **housing and husbandry conditions**.	Yes	Material and methods; Gene-Targeted Mice

Plants and microbes	Information included in the manuscript?	In which section is the information available? (Reagents and Tools Table, Materials and Methods, Figures, Data Availability Section)
**Plants:** provide species and strain, ecotype and cultivar where relevant, unique accession number if available, and source (including location for collected wild specimens).	Not Applicable	
**Microbes:** provide species and strain, unique accession number if available, and source.	Not Applicable	

Human research participants	Information included in the manuscript?	In which section is the information available? (Reagents and Tools Table, Materials and Methods, Figures, Data Availability Section)
If collected and within the bounds of privacy constraints report on age, sex and gender or ethnicity for all study participants.	Not Applicable	

Core facilities	Information included in the manuscript?	In which section is the information available? (Reagents and Tools Table, Materials and Methods, Figures, Data Availability Section)
If your work benefited from core facilities, was their service mentioned in the acknowledgments section?	Not Applicable	

Design

Study protocol	Information included in the manuscript?	In which section is the information available? (Reagents and Tools Table, Materials and Methods, Figures, Data Availability Section)
If study protocol has been **pre-registered, provide DOI in the manuscript**. For clinical trials, provide the trial registration number **OR** cite DOI.	Not Applicable	
Report the **clinical trial registration number** (at ClinicalTrials.gov or equivalent), where applicable.	Not Applicable	

Laboratory protocol	Information included in the manuscript?	In which section is the information available? (Reagents and Tools Table, Materials and Methods, Figures, Data Availability Section)
Provide DOI OR other citation details if **external detailed step-by-step protocols** are available.	Not Applicable	

Experimental study design and statistics	Information included in the manuscript?	In which section is the information available? (Reagents and Tools Table, Materials and Methods, Figures, Data Availability Section)
Include a statement about **sample size** estimate even if no statistical methods were used.	Yes	Material and methods; Statistical Analysis
Were any steps taken to minimize the effects of subjective bias when allocating animals/samples to treatment (e.g. **randomization procedure**)? If yes, have they been described?	Yes	Material and methods; Behavioral Assays
Include a statement about **blinding** even if no blinding was done.	Yes	Materials and methods; Behavioral Assays (All behavioral tests were performed by blinded investigators)
Describe **inclusion/exclusion criteria** if samples or animals were excluded from the analysis. Were the criteria pre-established?<br><br>If sample or data points were omitted from analysis, report if this was due to attrition or intentional exclusion and provide justification.	Yes	Material and methods; ECG and telemetry (one male WT mouse removed the transmitter lead and was dropped from all telemetry analysis)
For every figure, are **statistical tests** justified as appropriate? Do the data meet the assumptions of the tests (e.g., normal distribution)? Describe any methods used to assess it. Is there an estimate of variation within each group of data? Is the variance similar between the groups that are being statistically compared?	Yes	Material and methods; Statistical Analysis and appropriate Figure legends

Sample definition and in-laboratory replication	Information included in the manuscript?	In which section is the information available? (Reagents and Tools Table, Materials and Methods, Figures, Data Availability Section)
In the figure legends: state number of times the experiment was **replicated** in laboratory.	Yes	Figure legends
In the figure legends: define whether data describe **technical or biological replicates**.	Yes	Figure legends

Ethics

Ethics	Information included in the manuscript?	In which section is the information available? (Reagents and Tools Table, Materials and Methods, Figures, Data Availability Section)
Studies involving **human participants**: State details of **authority granting ethics approval** (IRB or equivalent committee(s), provide reference number for approval.	Not Applicable	
Studies involving **human participants**: Include a statement confirming that **informed consent** was obtained from all subjects and that the experiments conformed to the principles set out in the WMA Declaration of Helsinki and the Department of Health and Human Services Belmont Report.	Not Applicable	
Studies involving **human participants:** For publication of **patient photos**, include a statement confirming that consent to publish was obtained.	Not Applicable	
Studies involving experimental **animals**: State details of **authority granting ethics approval** (IRB or equivalent committee(s), provide reference number for approval. Include a statement of compliance with ethical regulations.	Yes	Material and methods, Gene-Targeted Mice.
Studies involving **specimen and field samples:** State if relevant **permits** obtained, provide details of authority approving study; if none were required, explain why.	Not Applicable	

Dual Use Research of Concern (DURC)	Information included in the manuscript?	In which section is the information available? (Reagents and Tools Table, Materials and Methods, Figures, Data Availability Section)
Could your study fall under dual use research restrictions? Please check biosecurity documents and list of **select agents and toxins** (CDC): https://www.selectagents.gov/sat/list.htm	Not Applicable	
If you used a select agent, is the security level of the lab appropriate and reported in the manuscript?	Not Applicable	
If a study is subject to dual use research of concern regulations, is the name of the **authority granting approval and reference number** for the regulatory approval provided in the manuscript?	Not Applicable	

Reporting

The MDAR framework recommends adoption of discipline-specific guidelines, established and endorsed through community initiatives. Journals have their own policy about requiring specific guidelines and recommendations to complement MDAR.

Adherence to community standards	Information included in the manuscript?	In which section is the information available? (Reagents and Tools Table, Materials and Methods, Figures, Data Availability Section)
State if relevant guidelines or checklists (e.g., **ICMJE, MIBBI, ARRIVE, PRISMA**) have been followed or provided.	Not Applicable	
For **tumor marker prognostic studies,** we recommend that you follow the **REMARK** reporting guidelines (see link list at top right). See author guidelines, under 'Reporting Guidelines'. Please confirm you have followed these guidelines.	Not Applicable	
For **phase II and III randomized controlled trials**, please refer to the **CONSORT** flow diagram (see link list at top right) and submit the CONSORT checklist (see link list at top right) with your submission. See author guidelines, under 'Reporting Guidelines'. Please confirm you have submitted this list.	Not Applicable	

Data Availability

Data availability	Information included in the manuscript?	In which section is the information available? (Reagents and Tools Table, Materials and Methods, Figures, Data Availability Section)
Have **primary datasets** been deposited according to the journal's guidelines (see 'Data Deposition' section) and the respective accession numbers provided in the Data Availability Section?	Not Applicable	
Were **human clinical and genomic datasets** deposited in a public access-controlled repository in accordance to ethical obligations to the patients and to the applicable consent agreement?	Not Applicable	
Are **computational models** that are central and integral to a study available without restrictions in a machine-readable form? Were the relevant accession numbers or links provided?	Not Applicable	
If publicly available data were reused, provide the respective **data citations in the reference list**.	Not Applicable	
